# Evaluating the Use of PROMs in Paediatric Orthopaedic Registries

**DOI:** 10.3390/children10091552

**Published:** 2023-09-14

**Authors:** Eleanor J. Morris, Kelly Gray, Paul J. Gibbons, Jane Grayson, Justin Sullivan, Anita B. Amorim, Joshua Burns, Marnee J. McKay

**Affiliations:** 1 Sydney School of Health Sciences, Faculty of Medicine and Health, The University of Sydney, Sydney 2006, Australia; eleanor.morris@health.nsw.gov.au (E.J.M.); jane.grayson@sydney.edu.au (J.G.); justin.sullivan@sydney.edu.au (J.S.); anita.amorim@sydney.edu.au (A.B.A.); marnee.mckay@sydney.edu.au (M.J.M.); 2 Sydney Children’s Hospitals Network, The Children’s Hospital at Westmead, Sydney 2145, Australia; paul.gibbons@health.nsw.gov.au; 3 Department of Health Sciences, Faculty of Medicine, Health and Human Sciences, Macquarie University, Sydney 2109, Australia; kelly.gray@mq.edu.au; 4 Sydney Children’s Hospitals Network, Paediatric Gait Analysis Service of New South Wales, Sydney 2145, Australia

**Keywords:** paediatric, PROMs, orthopaedic, registry, systematic review

## Abstract

Patient-reported outcome measures (PROMs) provide structured information on the patient’s health experience and facilitate shared clinical decision-making. Registries that collect PROMs generate essential information about the clinical course and efficacy of interventions. Whilst PROMs are increasingly being used in adult orthopaedic registries, their use in paediatric orthopaedic registries is not well known. The purpose of this systematic review was to identify the frequency and scope of registries that collect PROMs in paediatric orthopaedic patient groups. In July 2023, six databases were systematically searched to identify studies that collected PROMs using a registry amongst patients aged under 18 years with orthopaedic diagnoses. Of 3190 identified articles, 128 unique registries were identified. Three were exclusively paediatric, 27 were majority paediatric, and the remainder included a minority of paediatric patients. One hundred and twenty-eight registries collected 72 different PROMs, and 58% of these PROMs were not validated for a paediatric population. The largest group of orthopaedic registries collected PROMs on knee ligament injuries (21%). There are few reported dedicated orthopaedic registries collecting PROMs in paediatric populations. The majority of PROMs collected amongst paediatric populations by orthopaedic registries are not validated for patients under the age of 18 years. The use of non-validated PROMs by registries greatly impedes their utility and impact. Dedicated orthopaedic registries collecting paediatric-validated PROMs are needed to increase health knowledge, improve decision-making between patients and healthcare providers, and optimise orthopaedic management.

## 1. Introduction

Patient-reported outcome measures (PROMs) are tools that are designed to assess a patient’s perception of their health-related quality of life and their functional health status without interpretation from a medical professional [1,2]. Self-assessment, by means of a questionnaire, is considered the best method of evaluating patient-based outcomes, as any influence from a clinician or investigator is removed [2]. By assessing a patient’s subjective health experience and the consequence of any intervention [2], PROMs are an essential tool to understand the impact a condition has on an individual’s symptoms and disability [3]. PROMs are vital to shared clinical decision-making and patient-centred care as they provide key information regarding the natural history of conditions and the efficacy of interventions that can assist all healthcare stakeholders (patients, healthcare professionals/providers, and policymakers) facing healthcare decisions [4]. The broad utility and high importance of PROMs are reflected in their widespread adoption and standardised use amongst regulatory bodies, such as the US Food and Drug Administration and the European Medicines Agency, both of which mandate the use of PROMs to support labelling claims [5,6]. The use of PROMs has increased substantially in the field of orthopaedics over the last 20 years as the evidence for their importance has grown [1]. Since 2009, it has been mandatory to use PROMs to report outcomes for certain elective surgeries in the United Kingdom. The National Health Service publishes data from PROMs following orthopaedic surgical procedures to help drive improvements in surgical performance and service delivery [7].

Evidence of the increased use of PROMs is seen in the growing number of orthopaedic registries that have adopted PROMs [1]. Registries were first established in the fields of arthroplasty and trauma to monitor implant survival [1]. However, in recent decades, the utility of registries has been demonstrated by understanding patient characteristics, improving the timing and safety of intervention, and optimising public health decision-making [8]. If registries are large enough and include an adequate follow-up, they can provide an ideal platform for clinical trials, reducing resources required for prospective data collection [9]. Registry data can also be used to assist in answering questions that are not practical or ethical to address by randomised controlled trials [10]. By tracking health outcomes over time, it is possible to identify the under-utilisation of evidence-based practices and areas for improvement [11]. There is strong evidence that registry information can drive continuous improvements in patient outcomes and adherence to guideline-recommended care [10]. Registries, however, cannot achieve these goals without the inclusion of PROMs [8]. For example, in arthroplasty registries, the use of PROMs is now considered essential to determine a valid understanding of treatment success. Similarly, the improved survival rate in trauma registries has highlighted the need to collect PROMs to measure quality of life after injury [12].

Despite the importance of PROMs, there is little consistency in the use of PROMs in paediatric orthopaedics, and their use is infrequent compared to adult orthopaedics [2,13]. Furthermore, where PROMs are used, they are commonly not validated for paediatric populations [13,14]. If PROMs are not valid in the assessed population, they cannot be relied upon to measure the true impact of an intervention or inform healthcare decisions [14]. The standardised use of validated PROMs in paediatric orthopaedic registries is an essential step towards improving clinical care in paediatric orthopaedics [13,15]. Whilst PROMs orthopaedic registries are utilised in adult populations to improve the safety and efficacy of healthcare, in addition to strengthening communication and understanding between patients and healthcare providers, little is known about the use of PROMs in paediatric orthopaedic registries.

To ensure that PROM collection in paediatric orthopaedic registries is valid and useful in improving clinical understanding and care, it is crucial to identify gaps and weaknesses in the current state of PROM collection. It is vital to establish the current state of PROM collection by paediatric orthopaedic registries in order to highlight the most pressing issues and challenges facing this field of research and guide the future creation of registries. The aim of this systematic review is to achieve this goal by identifying the frequency and scope of registries that collect PROMs in paediatric orthopaedic patient groups and highlighting factors that need to be addressed to improve their utility.

## 2. Materials and Methods

This systematic review was performed following the guidelines for best practice in transparent, reproducible, and ethical reporting of systematic reviews (Preferred Reporting Items for Systematic Reviews and Meta-Analysis—PRISMA), and the protocol was registered (PROSPERO—CRD42021215364). Six electronic databases were searched from inception to 17 July 2023: Medline, Embase, Web of Science, Scopus, Cinahl, and Google Scholar. The search was developed with the assistance of an experienced librarian (KE) and tailored to each database using search terms that were a mix of database-controlled keywords, medical subject headings (MeSH), and the keywords p(a)ediatric, orthop(a)edic, registry and patient-reported outcome measures. The full search strategy is shown in Supplementary Text S1.

We included peer-reviewed, full-text, observational cohort, and case-control studies that included paediatric patients (<18 years), collected PROMs, had primary orthopaedic diagnoses, and included the use of a database or registry to collect PROMs. Patients were considered to have ‘primary orthopaedic diagnoses’ if the orthopaedic diagnosis was the primary reason for seeking treatment and if they were reviewed by an orthopaedic specialist. Studies were excluded if an English translation was unavailable, if they were limited to systematic reviews or published protocols, if they primarily focused on craniofacial orthopaedic diagnoses, or if they did not collect PROMs prospectively in the registry or database. Craniofacial diagnoses were excluded since they are included in the orthodontics and dentistry literature and not orthopaedics. Studies were grouped by the proportion of patients under the age of 18 years and according to their diagnostic inclusion. 

After removing duplicates, two reviewers (EM, KG) independently screened titles and abstracts and five reviewers (EM, KG, JG, JS, AA) independently screened full-text studies against the inclusion criteria using Covidence software (Veritas Health Innovation, Melbourne, Australia, 2023). Discrepancies between reviewers were resolved via discussion, with the support of a third review author (MM) if consensus was not reached. These discrepancies involved <9% of articles and were only related to the reason for exclusion. Of the studies included after full-text screening, each reference list was checked to identify other relevant studies for inclusion. No additional studies were identified using this method. 

### Data Extraction and Analysis

Using a standard form in Covidence, the data were extracted by one researcher (EM). The data extraction included: name of registry, scope of registry, country of registry, active years of registry, diagnostic criteria of included patients, age range of included patients, gender of included patients, PROMs used, time points of PROM collection, mode of PROM collection, sample size, type of study, nature of interventions examined, summary of findings of study, and how PROMs contributed to these findings. The scope was defined as ‘hospital’ if the registry collected data from a single hospital, ‘regional’ if the registry collected data from multiple hospitals, in a similar area, ‘national’ if a concerted effort was made to collect data from most, if not all, relevant hospitals/services in that country, and ‘international’ if data were collected from more than one country. 

The risk of bias of all included studies was assessed using the Newcastle Ottawa Quality Assessment Scale (NOS) for cohort or case control studies, using Covidence software, by EM and KG. This scale was used because it was developed specifically for cohort and case control studies, which were the two types of studies that this systematic review identified. The criteria used by NOS to assess quality are provided in Supplementary Text S2. Studies with NOS scores of 0–3, 4–6, and 7–9 were considered as low, moderate, and high quality, respectively [16]. 

## 3. Results

### 3.1. Literature Search

**Figure 1:**
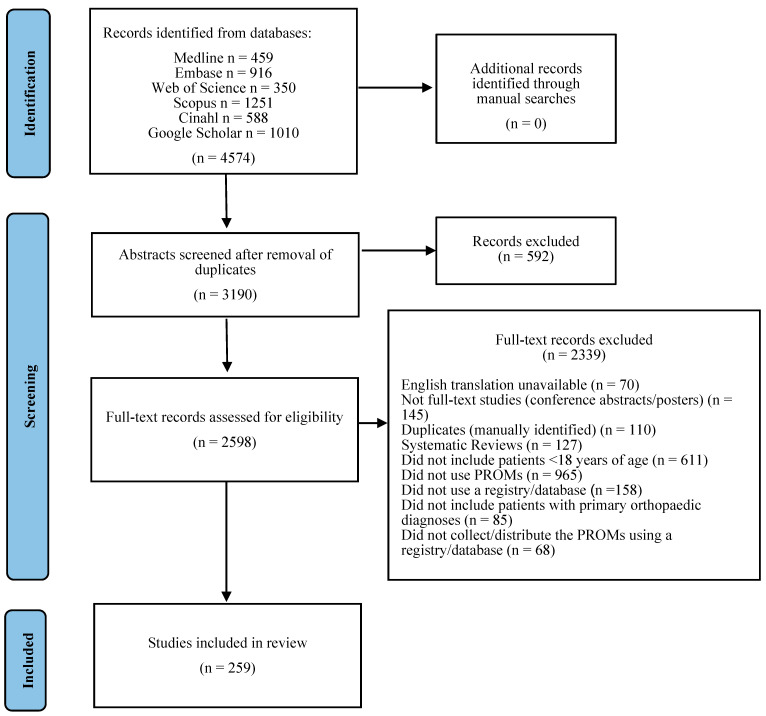
PRISMA flow chart of study selection.

The process of screening is summarised in the PRISMA flow diagram (Figure 1). A total of 4383 studies were identified through the search strategy. After the automatised removal of duplicates, 3011 studies remained. The titles and abstracts of the 3011 studies were screened, with 467 excluded due to not meeting the inclusion criteria. The remaining 2544 studies were then assessed for full-text eligibility by application of the inclusion and exclusion criteria. Covidence software allows only a single reason for exclusion, however, some studies would be excluded for more than one reason. The exclusion reason was chosen according to the order displayed in Figure 1. Of the 2339 studies that were excluded, 965 did not use PROMs, 611 did not include patients under the age of 18 years, 145 were not full-text studies (conference abstracts or poster presentations), 158 did not use a registry or database, 127 were systematic reviews, 110 were duplicates that had not been previously identified, 85 did not include patients with primary orthopaedic diagnoses, 70 did not have an available English translation, and 68 did not collect PROMs prospectively using a registry or database. After this assessment, 259 (10%) full-text studies were included in the analysis. 

### 3.2. Description of Studies and Risk of Bias

Of the 259 included studies, the majority were observational cohort studies, with the exception of 91 case-control studies. The style and purpose of the studies differed greatly, as seen in Table 1, Table 2, Table 3 and Table 4. The risk of bias score for all studies, using the NOS for cohort or case control studies, is provided in the final column of Table 1, Table 2, Table 3 and Table 4. All studies achieved scores of high quality (7–9), with the exception of five studies, which were rated as moderate quality. Four studies scored 6 [17,18,19,20,21] and one study scored 5 [21]. These studies were considered to have a higher risk of bias due to inadequate follow-up and lack of comparability of the cohort. The remaining studies (98%) were rated as having a low risk of bias. Complete details of the risk of bias scores for all included studies are provided in Supplementary Text S2.

### 3.3. Type of PROMs

The registries used 72 different PROMs, including 24 generic, 8 hip pathology-specific, and 14 knee-pathology-specific (Table 5). Amongst these 72 PROMs, 42 (58%) did not include any paediatric validation, and 7 (10%) included validation limited to those 16 years and over. In the 3 exclusively paediatric registries, all PROMs used were validated for paediatric populations, and amongst the 27 majority paediatric registries, 61% of the PROMs used were validated for those under 18 years of age. Regarding PROM collection frequency, 21% of the registries collected PROMs as a one-off, and the remainder collected them at multiple time points. The three most common PROM collection time points were pre-surgery, one-year post-surgery, and two years post-surgery, however, there was great variation across all registries.

### 3.4. Registries

**Figure 2:**
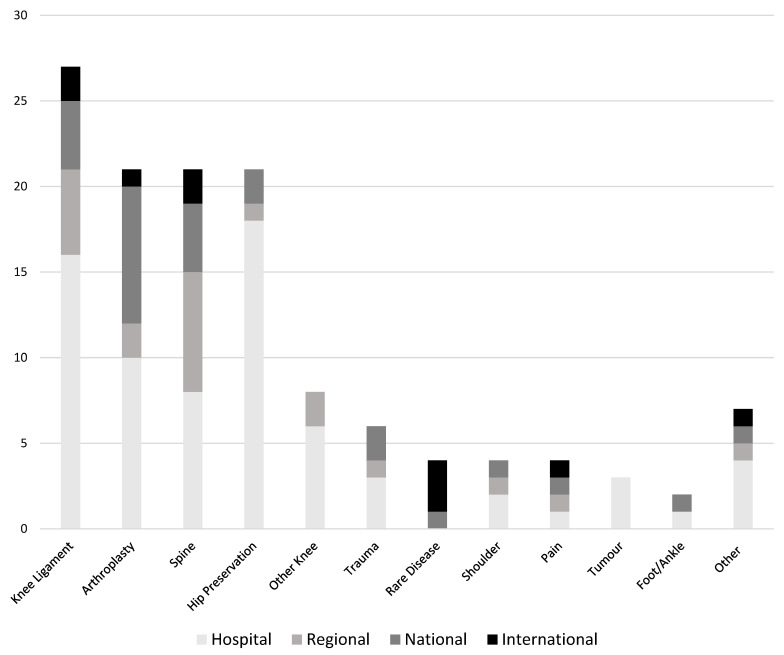
Scope of registries that include patients under the age of 18 years.

Overall, 128 unique registries that included patients under the age of 18 years in their reported data sets were identified. There were three registries that included exclusively paediatric patients (Table 1), 27 registries that included a majority (>50%) of paediatric patients (Table 2), 16 registries that included a minority (33–50%) of paediatric patients (Table 3), and 82 registries that included a small minority (<33%) of paediatric patients. (Table 4). There were 27 knee ligament registries, 21 arthroplasty registries, 21 spine registries, and 21 hip preservation registries (Table 6). The scope of registries ranged from single hospital-based to international, with 56% (n = 72) of all included registries limited to a single-hospital scope. We identified 21 regional registries, 25 national registries, and 10 international registries. (Figure 2).

#### 3.4.1. Knee Ligament Registries

Of the 27 knee ligament registries that included patients under the age of 18 years, 16 were hospital-based registries, and 4 were national registries: the Danish, Swedish, Norwegian, and New Zealand Knee Ligament Registries [108,165,184,190]. One registry was a majority paediatric hospital-based registry that used only PROMs validated for those under 18 years (Pediatric–International Knee Documentation Committee (Pedi-IKDC) and Children’s Health Questionnaire(CHQ)) [29]. The remaining 26 registries were minority paediatric but had notably larger proportions of patients aged under 18 years compared to the arthroplasty registries (Table 6). These registries used 23 PROMs, including 11 generic PROMs and 12 knee-specific PROMs. The two most used knee-specific PROMs were the Knee Injury and Osteoarthritis Outcome Score (KOOS), which is validated for those 16 years and over, and the International Knee Documentation Committee (IKDC), which is not validated for paediatrics.

#### 3.4.2. Lower Limb Arthroplasty Registries

The lower limb arthroplasty registries included a small minority of paediatric patients, with the exception of one [28]. Most were hip arthroplasty registries, of which two were national registries, with the majority being limited to a single-hospital scope [142,153]. There were three that included hip, knee, and ankle arthroplasties in one registry [143,145] There were nine anatomy-specific and eight generic PROMs used by these registries (Table 5). The most commonly used were the Visual Analogue Scale (VAS), European Quality of Life—5 dimensions (EQ5D), and the Western Ontario and McMaster Universities Osteoarthritis Index (WOMAC), which were each used in four different registries. Of these, the WOMAC is not validated for paediatrics, the EQ5D is validated for those 16 years and over, and the VAS is validated for paediatric patients from the age of five years.

#### 3.4.3. Spine Registries

There were 21 spine registries that included patients under the age of 18 years. Only 1 was exclusively paediatric [22], and a further 15 reported a majority of paediatric patients (Table 2). The most frequently used PROM was the Scoliosis Research Society Questionnaire (SRS) (various versions), which has been validated for the paediatric population from the age of 10 years. In both majority and minority paediatric registries, this PROM was occasionally used amongst participants younger than 10 years [22,84]. Other PROMs used and validated for paediatric patients included the Early-Onset Questionnaire (EOSQ24) and the Caregiver Priorities Child Health Index of Life with Disabilities (CPCHILD) [55,61]. Similar to the SRS, the Short Form 12 and 36 (SF12, SF36), the Body Image Disturbance Questionnaire (BIDQ), and the European Quality of Life 5 Dimensions 3 Levels (EQ5D3L) were all used in patients below the age of their paediatric validation range, and the Oswestry Disability Index (ODI) was used in spine registries despite not being validated for those under the age of 18 years [93,94,227].

#### 3.4.4. Hip Preservation Registries

We identified 21 hip preservation registries that included patients under the age of 18 years. A total of 2 of these had a majority of paediatric patients [30], and 18 were hospital-based. These 21 registries used 11 PROMs, including 8 hip-specific PROMs. Of these, only the Hip Outcome Score (HOS) was validated for patients under 18 years and utilised in 5 of the 21 hip preservation registries (Table 1, Table 2, Table 3, Table 4 and Table 5).

## 4. Discussion

This review highlights the paucity of PROM collection amongst paediatric patients by orthopaedic registries; specifically, only three dedicated paediatric registries collect PROMs in paediatric orthopaedic populations. There were an additional 125 orthopaedic registries that included both adults and paediatric patients, with 98 of these registries including a minority of individuals aged under 18 years. Of all studies reporting these registries, 98% were of high quality, with a low risk of bias. Registries that collect PROMs typically establish a structure for studies that avoids a number of risks associated with single studies, including bias in-patient selection, comparability of cohorts, prospective data collection, and duration of follow-up. Whilst these concerns are usually not an issue for a well-designed registry, the challenge of an adequate response rate, which was the NOQAS criterion most frequently not met by the studies in this review, can be a significant concern.

The importance of well-designed and well-maintained registries that minimise loss to follow-up has been widely established in adult populations [1]. Such high-quality registry data have resulted in improved models of care in a number of health specialties. Some examples include accelerated ulcer healing time, attributed to the Swedish Ulcer Registry [345], and established causes of mortality associated with rheumatoid arthritis [346]. Furthermore, diabetes registries have improved attendance at appointments and compliance with treatment regimens [347] and the Australian Breast Device Registry detected three devices with high complication rates, which were subsequently removed by the Therapeutic Goods Administration, resulting in reduced national revision rates [348]. Likewise, in orthopaedics, data from the Australian Joint Replacement Registry identified high revision rates associated with the ASR™ Hip Resurfacing System, leading to a substantial reduction in their use and an overall reduction in hip and knee arthroplasty revisions since the registry has been in operation [349]. The Victorian Orthopaedic Trauma Outcomes Registry identified key factors in demographics and injury management affecting return to work and mortality in those under 65 years who sustain a hip fracture [245,246]. 

The second largest proportion of registries identified in this review were arthroplasty registries that consistently use PROMs not validated for use in people aged under 18 years. Whilst the average age of patients undergoing arthroplasty was greater than 70 years in the early 1990s, in recent years, the average age has decreased, and future projections indicate that it will continue to do so [350]. In light of the historically older age, it is not surprising that arthroplasty registries were not established with paediatrics in mind [350]. However, given the documented increased frequency of paediatric arthroplasty [351,352,353], it is now essential that registries accommodate paediatric patients. The majority of the remaining orthopaedic registries identified in this review concern specific diagnostic groups such as knee ligament reconstruction, hip preservation procedures, spine surgery, and trauma. It is paramount that registries for these diagnostic groups collect validated PROMs for the age range of included children so that information gathered can be utilised to improve the clinical course of these conditions and gauge the efficacy of interventions [13].

One barrier to the inclusion of paediatric-validated PROMs in orthopaedic registries may be the limited number of appropriate PROMs available for specific diagnostic groups. Currently, the only hip-specific PROM with paediatric validation is the Hip Outcome Score, which is validated for those aged 13 years and over [305]. A systematic review of hip PROMs used in older paediatric patients did not comment on whether the PROMs used were validated for the reported age group [354]. Likewise, the lack of adequate PROMs is a significant challenge shared by rare disease diagnostic groups with orthopaedic involvement. The use of non-validated custom questionnaires by many of the rare disease registries highlights the inadequacy of existing validated PROMs for their purposes [21,101,102]. A lack of validated PROMs significantly reduces the extent to which orthopaedic registries can capture relevant and valid information to ultimately improve healthcare efficacy and safety [13,355].

This review shows that when paediatric-validated PROMs are available, they are rarely used by orthopaedic registries that include paediatric patients [356,357]. A challenge in using paediatric-validated PROMs in registries that include both adults and paediatric patients may be the increased burden of customising PROM delivery according to age [3]. This was apparent in the knee ligament registries, which overwhelmingly used the KOOS [112,172] and/or the IKDC [119,358], and not the KOOS-child, validated from 16 years of age, or the Pedi-IKDC, which is validated and recommended for those under 18 years of age [315,359]. Improved registry design to collect valid data from all patients that can be utilised to understand the natural history and surgical outcomes from childhood through to adulthood is required. The burden of integrating paeditric and adult versions of a PROM in the same registry can be overcome with digital platforms, such as research electronic data capture (REDCap) [360], which can automatically distribute age-appropriate validated PROMs. 

Another possible reason for registries not using validated paediatric PROMs when available may be the challenge of comparing scores between paediatric and adult-version PROMs [3]. This again can be overcome by using paediatric and adult versions of the same PROM that have published equivalency scores [359]. By doing so, such registries would improve the understanding of orthopaedic conditions, and the impact of interventions as paediatric patients transition into adulthood. The integration of scores between two different PROMs remains a substantial challenge. Further research to establish the clinical and statistical relationship between the most appropriate paediatric and adult PROM will only be possible if appropriate validated PROMs are used in these registries. 

The findings of this review point to two key actions that can be undertaken to improve PROM collection by orthopaedic registries. Firstly, for adult registries that include participants under the age of 18 years, accommodations must be made for these younger participants to ensure the data that are collected are valid and useful. Secondly, there is a need for further dedicated paediatric orthopaedic registries that collect PROMs in order to answer future questions concerning paediatric orthopaedic conditions and interventions. Such actions may be accelerated if policies are introduced by health services that require more uniform PROM collection amongst orthopaedic populations such as has been seen in arthroplasty registries [4]. Furthermore, insistence on the use of validated PROMs by journals would result in registries no longer using non-validated tools. These changes have the potential to transform the scope and quality of paediatric orthopaedic research. Such improvements would increase the understanding of how orthopaedic conditions affect children and raise the standard of care provided to such children.

We acknowledge the limitations of this review. First, our search criteria included any registry that included patients under 18 years of age. This resulted in a large number of registries that included a very small proportion of paediatric patients, including a number of registries that included one or two 17-year-olds. However, we attempted to make this issue transparent by grouping the registries by the proportion of paediatric patients they included (Table 1, Table 2, Table 3 and Table 4). Second, the exclusion of craniofacial orthopaedic diagnoses was undertaken due to a large overlap with dental medicine publications, as these were considered too far removed from the common understanding of paediatric orthopaedics. Further reviews examining the relevance of these articles may be indicated. Third, we acknowledge there may be registries in existence that collect validated PROMs in paediatric orthopaedic populations but have not yet published their findings and were, therefore, not included in this systematic review.

## 5. Conclusions

Currently, there are only three reported registries with publications that have been established to collect PROMs in paediatric orthopaedic patients, though many adult orthopaedic registries include the collection of PROMs in paediatric patients. Comparing this small number to the frequency of adult orthopaedic registries highlights the paucity of paediatric orthopaedic registries that collect PROMs. Given that these three registries report data collected since 2000, it is apparent that this is an area of clinical research that has been slow to change. The lack of systematic collection of validated PROMs in paediatric orthopaedics through registries means that the paediatric orthopaedic literature is largely dependent on clinician-reported outcomes and individual studies. This reduces the understanding of conditions and treatment impact from the perspective of the patient. As a result, the research findings may be limited by patient numbers and a narrower scope of investigated questions. In contrast, registries that collect PROMs provide essential information about the course of clinical conditions and interventions from the patient’s perspective, ultimately promoting patient-centred care and shared decision-making. Therefore, if we are to better understand health conditions, assess interventions and improve the quality and safety of care in paediatric orthopaedics, registries must be established and must use validated PROMs in their target populations. An investment in infrastructure to support the collection of PROMs by registries in paediatric orthopaedics is needed from health service providers and policymakers. Such changes will allow health outcomes to be assessed in children and tracked as children grow into adults.

## Figures and Tables

**Table 1:** Registries reporting exclusively paediatric patients.

Registry	Scope: Hospital/Regional/National/International (Nation)	Years Active	Publications (Type of Study)	Diagnostic Inclusion	Number of Patients in Publication (% of Registry)	Patient Age Range (Years) in Publication (Mean and SD or Median)	PROMs Used	Frequency of PROM Collection	Risk of Bias (0–9)
Spine Registries									
Multi-Center Spine Registry	Regional (USA)	Not stated (PD: 2000–2018)	Qiu et al. [22] (OC)	Idiopathic scoliosis and posterior spinal fusion	82	8–16 (Mean: 11.7, SD: 1.2)	SRS-22✗	Once	7
Trauma Registries									
Hospital Trauma and Psychology Database	Hospital (UK)	Not stated (PD: 2013–2018)	Messner et al. [23] (OC)	Open lower limb trauma	32	4–17	PedsQL✓ CRIES✗	Once	8
Other Registries									
Congenital Upper Limb Differences (CoULD) Registry	Regional (USA)	2014–present	Bae et al. [24] (OC) Daley et al. [25] (OC)	Congenital upper extremity difference	301 (51%) 260	2–17 (Median: 7.8) (Mean: 8, SD: 4)	PODCI✓ PROMIS✓	Once	8 7
			Wall et al. [26] (OC)		375	5–17 (Mean: 11)			8
			Wall et al. [27] (CC)		120	2–17 (Mean: 6.5)			7

Key: ✓ = PROM validated for age range of study, ✗ = age range of study is outside validated range of PROM, CRIES: Children’s Revised Impact of Event Scale, CC: case control study, OC: observational cohort study, PD: published data, PedsQL: Pediatric Quality of Life Inventory TM, PODCI: Pediatric Outcomes Data Collection Instrument, PROMIS: Patient-Reported Outcomes Measurement Information System, SRS: Scoliosis Research Society, UK: United Kingdom, USA: United States of America.

**Table 2:** Registries reporting majority paediatric patients (>50%).

Registry	Scope: Hospital/Regional/National/International (Nation)	Years Active	Publications (Type of Study)	Diagnostic Inclusion	Number of Patients in Publication (% of Registry)	Patient Age Range (Years) in Publication (Mean and SD or Median)	PROMs Used	Frequency of PROM Collection	Risk of Bias (0–9)
Arthroplasty Registries									
Hospital Total Joint Registry	Hospital (USA)	Not stated (PD: 1998–2016)	Pallante et al. [28] (OC)	Total hip arthroplasty	78	11–20 (Mean: 17)	mHHS✗	Once	8
Knee Ligament Registries									
Hospital ACL Database	Hospital (USA)	(PD: 2007–2009)	Boykin et al. [29] (OC)	ACL rupture	135	13–17 (Median: 15)	PediIKDC✓ CHQ✓	Once	8
Hip Preservation Registries									
Hospital Hip Preservation Registry	Hospital (USA)	Not stated (PD: 2010–2014)	Nwachukwu et al. [30] (OC)	Arthroscopic treatment of FAI	47	(Mean: 16.5)	iHOT-33✗ mHHS✗ HOS✓	Before surgery, after surgery: 12 months	8
Hospital FAI Registry	Hospital (USA)	Not stated	Serbin et al. [31] (CC)	Surgical treatment of FAI	81	10–20	mHHS✗ HOOS✗	Before surgery, after surgery: 12, 24 months	7
Spine Registries									
Multi-Center Scoliosis Registry (Harms Study Group)	Regional (USA)	1995–present (PD: 1997–2016)	Bastrom et al. [32] (CC)	AIS	1193	(Mean: 15, SD: 2)	SRS-7✓ SRS-24✓ SRS-22✓ SRS-22r✓	Before surgery, after surgery: 12, 24, 60, 120 months	8
			Bastrom et al. [33] (OC)	Posterior spinal fusion	1695	(Mean: 14.7, SD: 2)			8
			Bastrom et al. [17] (OC)	Surgical correction of AIS	829	Not stated			6
			Bastrom et al. [34] (OC)	AIS with an operative COBB range	584	10–21 (Mean: 14.7, SD: 2)			7
			Benes et al. [35] (OC) Bennett et al. [36] (OC)	Posterior spinal fusion and infection AIS	47 99	(Mean: 15, SD: 2) (Mean: 14, SD: 2.1)			7 7
			Bennett et al. [37] (CC)	AIS	1020	(Mean: 14, SD: 2.1)			7
			Buckland et al. [38] (OC)	Surgical correction of AIS	2210	(Mean: 14.7, SD: 2.1)			8 8
			Hughes et al. [39] (CC)	AIS	916	(Mean: 14.3, SD: 2.1)			7
			Jain et al. [40] (OC)	AIS	685	(Mean: 14.7, SD: 2.2)			7
			Kelly et al. [41] (OC)	Surgical correction of AIS	1281 (44%)	10–22 (Mean: 14.6)			7
			Lark et al. [42] (CC)	AIS	150	(Mean: 15, SD: 2)			8
			Lonner et al. [43] (OC)	AIS	1031	10–21			7
			Louer et al. [44] (OC)	AIS	51	(Mean: 14/15)			7
			Newton et al. [45] (OC) Newton et al. [46] (CC)	Major thoracic scoliosis Thoracic scoliosis	174 474	10–21 (Mean: 14.5, SD: 2.1 at surgery, mean: 25, SD: 2.3 at follow-up) 8–18			7 8
			Ohashi et al. [47] (CC)	Major thoracic AIS	405	10–21 (Mean: 14.4, SD: 2.1)			8
			Phillips et al. [48] (OC)	AIS with primary structural thoracolumbar curves	139	(Mean: 15.2, SD: 2)			8
			Schulz et al. [49] (OC)	AIS	106	(Mean: 14.5, SD: 2)			7
			Segal et al. [50] (CC)	AIS	225	(Mean: 14.5)			7
			Singla et al. [51] (CC) Stone et al. [52] (OC)	AIS AIS	74 3686	(Mean: 14.2) (Mean: 14.5, SD: 2.2)			7 7
			Upasani et al. [53] (OC)	AIS	49	(Mean: 14.2)			7
Multi-Center CP Spine Registry	Regional (USA)	Not stated (PD: 2008–2015)	Badin et al. [54] (CC) Eguia et al. [55] (OC)	Posterior spinal fusion (with CP) Posterior spinal fusion (with CP)	222	(Mean: 14, SD: 3) (Mean: 14, SD: 2.7)	CPCHILD✓	Before surgery, after surgery: 12, 24, 60 months	7 7
			Jain et al. [56] (OC)	CP	212	8–20 (Mean:14, SD: 2.6)			7
			Miller et al. [57] (OC)	Posterior spinal fusion (with non-ambulatory CP)	157	Not stated: <21			8
			Miyanji et al. [58] (OC)	CP and scoliosis	203	(Mean: 13.5, SD: 2.64)			7
			Vivas et al. [59] (OC)	Posterior spinal fusion (with CP)	218	(Mean: 14.2)			8
Paediatric Spine Study Group (Previously: Growing Spine Study Group and Children’s Spine Study Group)	International	Not stated (PD: 1997–2018)	Bauer et al. [60] (CC)	EOS	302	Not stated	EOSQ-24✓	Before surgery, after surgery: 24 months, end of treatment	7
			Campbell et al. [61] (CC) Gomez et al. [62] (OC)	EOS Congenital scoliosis	503 53	(Mean: 5.6, SD: 3.7) 1–11			7 7
			Heffernan et al. [63] (CC)	EOS	960	(Mean: 5.8/6.1)			8
			Helenius et al. [64] (CC)	Skeletal dysplasias	33 (6%)	1–10 (Mean: 5.3/5.4)			9
			Helenius et al. [65] (CC) Henstenburg et al. [66] (OC)	Severe and moderate EOS EOS	80 (14%) 66	1–9 (Mean: 5.4/5.3) 0–6			9 8
			Matsumoto et al. [67] (CC)	EOS	155	(Mean: 12.5, SD: 2.1)			8
			Matsumoto et al. [68] (CC)	EOS	91	(Mean: 2.1, SD: 1.2)			7
			Matsumoto et al. [69] (OC)	EOS	121	4–17 (Mean: 10.4, SD: 0.2)			9
			Matsumoto et al. [70] (CC)	SMA and EOS	74	2–12 (Mean: 7.6, SD: 2.3)			8
			Nossov et al. [71] (CC)	EOS	329	0–10			8
			Ramirez et al. [72] (OC)	EOS	30	2.7–9 (Mean: 5.3, SD: 2.6)			7
			Ramo et al. [73] (OC)	EOS	610	0–17(Mean: 6.1, SD: 3.8)			7
			Roye et al. [74] (OC)	EOS	443	12–23 (Mean: 14.9 SD: 1.8)			8
			Roye et al. [75] (CC)	EOS	325	(Mean: 6.4, SD: 2.5)			7
			Saarinen et al. [76] (CC) Shaw et al. [77] (OC)	EOS EOS treated with distraction instrumentation	88 150	(Mean: 7.4/7) (Mean: 7, SD: 2.6)			7 8
			Verhofste et al. [78] (CC)	AMC and EOS	57	(Mean: 6.2/6.4)			7
Spinal Deformity Study Group Registry	Regional (USA)	Not stated (PD: 2003–2007)	Carreon et al. [79] (OC)	Idiopathic scoliosis	887	10–18	SRS-22✗ SRS-30✗ SAQ✓	Before surgery, after surgery: 12, 24, 60 months	7
			Crawford et al. [80] (CC)		264	(Mean: 14.7/14.8)			7
			Fletcher et al. [81] (CC)		214	(Mean: 14.5, SD: 1.8)			8
			Landman et al. [82] (OC)		1433	Not reported			7
			Luhmann et al. [83] (CC)		101	(Mean: 15.8/16/15.9)			8
			Sieberg et al. [84] (OC)		260	8–21 (Mean: 14.35, SD: 2.23)			8
			Roberts et al. [85] (CC)		744	(Mean: 14/15.2)			7
			Sanders et al. [86] (CC)		477	(Mean: 13.97)			7
			Theologis et al. [87] (CC)		461	10–18			7
			Zebracki et al. [88] (OC)		45	(Mean: 16.5/15.1)			7
International Spine Registry	International	Not stated	Djurasovic et al. [89] (CC)	Idiopathic scoliosis	1510	(Mean: 14.53/15.12)	SRS22r✓	Before surgery, after surgery	7
Regional West Africa Spine Database	Regional (Ghana)	Not stated (PD: 2012–2013)	Nemani et al. [90] (OC)	AIS and traction	29	(Mean: 14, SD: 5)	SRS-22✓	Before surgery, after surgery: 1.5 months	7
Hospital Spine Registry	Hospital (Italy)	Not stated (PD: 2003–2009)	Negrini et al. [91] (CC)	Idiopathic scoliosis, COBB angle >45°, and refusal of surgical intervention	28 (0.4%)	(Mean: 14, SD: 1.8)	SRS-22✓	Once (end of treatment)	8
Multi-Centre Spine Registry	Regional (Canada)	Not stated (PD: 2009–2012)	Miyanji et al. [92] (CC)	Minimally invasive surgery for AIS	46	14–20 (Mean: 16.8)	SRS-22r✓	Before surgery, after surgery: 24 months	8
Hospital Spondylo-listhesis Registry	Hospital (Canada)	(PD: 2002–2009)	Bourassa-Moreau et al. [93] (CC)	Spondylolisthesis	34	7–20	SRS-22r✗ SF12✗	Before surgery, after surgery	7
Hospital AIS Registry	Hospital (USA)	(PD: 2016–2017)	Diebo et al. [94] (OC)	AIS	47	10–25 (Mean: 15, SD:3)	Srs30✓ BIDQ✓		7
Hospital Surgical Spine Database	Hospital (USA)	(PD: 2002–2012)	Godzik et al. [95] (CC)	Chiara malformation and AIS	41	(Mean: 14, SD:6)	SRS-22✓, -24✓, -29✓, -30✓	Before surgery, after surgery: 24 months	7
Hospital Spine Registry	Hospital (The Netherlands)	2014–present	Mens et al. [96] (OC)	AIS	144	(Mean: 15, IQR: 14–17)	SRS22r✓ EQ5D3L✗ ODI✗ NRS✓	Before surgery, after surgery: 24 months	7
Hospital Spine Registry	Hospital (China)	(PD: 2012–2014)	Zhu et al. [97] (CC)	AIS	45	(Mean: 16.5/15.1)	SRS22✓	Once	7
Hospital Congenital Scoliosis Database	Hospital (USA)	2006–present (PD: 2016–2017)	Li et al. [98] (OC)	Congenital scoliosis	98	0–18	EOSQ-24✓ SRS-22✓	Once	7
Hospital AIS Surgery Database	Hospital (USA)	(PD: 2016–2019)	Thomas et al. [99] (OC)	AIS	48	(Mean: 14.9, SD: 1.9)	SRS22✓	Before surgery, after surgery: 6, 24 months	7
Rare Disease Registries									
Australian Rett Syndrome Database	National (Australia)	1993–present (PD: 2000–2006)	Downs et al. [100] (OC)	Scoliosis	102 (33%)	4–24 (Mean: 13.1/15.2)	Modified parent-report WeeFIM✓ RS: SSI✓ RSBQ✓	Every 2 years	7
German Austrian DMD Registry	International (Germany and Austria)	Not stated (PD: 2017–2018)	Schorling et al. [101] (OC)	DMD	351 (24%)	60% < 16	Custom questionnaire✗	Once	8
Cure SMA Registry	International	1996–present, (PD: 2017–2018)	Belter et al. [21] (OC)	SMA	2017: 695 (10%) 2018: 796 (11%)	0–78 (Median: 11)	Custom questionnaire✗	Twice	6
Morquio Registry	International	(PD: 1998–2006)	Montano et al. [102] (OC)	MPS Morquio	326	1–73 (65% < 18)	Custom questionnaire✗	Once	8
Pain Registries									
Hospital Analgesia Registry	Hospital (USA)	Not stated (PD: 2003–2006)	Ganesh et al. [103] (OC)	Continuous CPNB for post-op analgesia following orthopaedic surgery	217	4–18 (Mean: 13.7, SD: 3.4)	vNRS✗	Ongoing during admission	7
Multi-Center Medical Record Pain Database	Regional (USA)	Not stated (PD: 2012–2019)	Zhang et al. [104] (CC)	Posterior spinal fusion for AIS	682	(Mean: 14)	NRS✓ VAS✓	Variable between sites	7
Other registries									
Motion Analysis Laboratory Database	Hospital (USA)	Not stated (PD: 1994–2013	McMulkin et al. [105] (CC)	Cerebral palsy and femoral derotation osteotomy	133	4–20	PODCI✓	Before surgery, after surgery	8
			Schwartz et al. [106] (OC)	Cerebral palsy	135	3–44	GFAQ✓		8
Other Knee Registries									
Hospital Osteochondral Allograft Registry	Hospital (USA)	Not stated (PD: 2004–2017)	Gilat et al. [107] (CC)	Osteochondral allograft transplant of the knee	46	(Mean: 16.8, SD: 1.3)	IKDC✗ Lysholm✗ KOOS✗ WOMAC✗ SF12✓	Before surgery, after surgery	7

Key: ✓ = PROM validated for age range in study, ✗ = age range of study is outside validated range of PROM, ACL: anterior cruciate ligament, AIS: adolescent idiopathic scoliosis, BIDQ: Body Image Disturbance Questionnaire, CC: case control study, CHQ: Child Health Questionnaire^TM^, CP: cerebral palsy, CPCHILD: Caregiver Priorities and Child Health Index of Life with Disabilities, CPNB: continuous peripheral nerve blockade, DMD: Duchenne muscular dystrophy, EOS: early-onset scoliosis, EOSQ-24: Early-Onset Scoliosis Questionnaire, FAI: femoro-acetabular impingement, GFAQ: Gillette Functional Assessment Questionnaire, HOS: Hip Outcome Score, iHOT-33: International Hip Outcome Tool, IKDC: International Knee Documentation Committee, KOOS: Knee Injury and Osteoarthritis Outcome Score, mHHS: Modified Harris Hip Score, MPS: mucopolysaccharidosis, OC: observational cohort study, PD: published data, PediIKDC: Pediatric Version International Knee Documentation Committee, PODCI: Pediatric Outcomes Data Collection Instrument, RSBQ: Rett Syndrome Behaviour Questionnaire, RS: SSI: Rett Syndrome: Symptom Severity Index, SAQ: Scoliosis Appearance Questionnaire, SF12: Short Form-12, SMA: spinal muscular atrophy, SRS: Scoliosis Research Society, USA: United States of America, VAS: Visual Analogue Scale, vNRS: Verbal Numerical Rating Scale, WeeFIM: Functional Independence Measure (Child version), WOMAC: Western Ontario and McMaster Universities Osteoarthritis Index.

**Table 3:** Registries reporting a minority of paediatric patients (33–50%).

Registry	Scope: Hospital/Regional/National/International (Nation)	Years Active	Publications (Type of Study)	Diagnostic Inclusion	Number of Patients in Publication (% of Registry)	Patient Age Range (Years) in Publication (Mean and SD or Median)	PROMs Used	Frequency of PROM Collection	Risk of Bias (0–9)
Knee Ligament Registries									
New Zealand ACL Registry	National (New Zealand)	2014–present	Fausett et al. [108] (OC) Rahardja et al. [109] (OC) Tiplady et al. [110] (OC)	ACLR	5345 (56%) 1844 1466	8–70 (Mean: 28, SD: 10) 15–20	KOOS✗ MARS✗	Before surgery, after surgery: 6, 12, 24 months	7 8 8
MOON ACL Database	Regional (USA)	2002–present	Dunn et al. [111] (OC)	ACL injury	525 (78)	(Mean: 26, SD: 11)	KOOS✗ MARS✗ SF36✗ IKDC✗ RTS✗	Before surgery, after surgery: 24 months.	8
			#Failla et al. [112] (CC)		1995	(Mean: 24.3, SD: 10)			8
			#Magnussen et al. [113] (OC)		713/950	(Median: 23 IQ: 17–35)			9
			Mather et al. [114] (OC)		988	(Mean: 26, SD: 11)			8
			Ramkumar et al. [115] (OC)		3202 (100%)				8
			Wright et al. [116] (OC)		273	11–54 (Mean: 24, median: 23)			8
Hospital ACL Registry	Hospital (Norway)	(PD:1987–1994)	Lindanger et al. [117] (CC)	ACL injury		14–47 (Mean: 22)	RTS✗	At follow-up (unspecified)	9
Delaware Oslo ACL Registry	International	2007–2012	#Failla et al. [112] (CC)	ACL injury	192 (64%)	13–60 (Mean: 24.7, SD: 9)	KOOS✗ IKDC✗	Before surgery, after surgery: 24 months.	8
			#Grindem et al. [118] (CC)		84	16–40 (Mean: 25.3, SD: 7.2)			8
Hospital ACL Registry	Hospital (USA)	(PD: 2009–2013)	Nwachukwu et al. [119] (OC) Nwachukwu et al. [120] (OC) Nwachukwu et al. [121] (OC) Randsborg et al. [122] (OC) Rauck et al. [123] (CC)	ACL injury	231 232 294 2042 (70%) 53/143	(Mean: 26.7, SD: 12.5) 13–63 (Mean: 26.7, SD: 12.5) (Mean: 25.5) (Mean: 30, SD: 12) (Mean: 16)	IKDC✗ Lysholm✗ Tegner✗ MARS✗ SF12✗ RTS✗	Before surgery, after surgery: 6,12,24,60 months	8
									7
									7
									7 7
Swedish ACL Rehab Registry	Regional (Sweden)	2009–present	Hamrin Senorski et al. [124] (OC)	ACL injury	157	15–30 (Mean: 20, SD: 3)	KOOS✗ Tegner✗ PAS✗ K-SES✗	After surgery: 2.5, 4, 8, 12, 18, 24 months	8
			Sundemo et al. [125] (CC)	ACL injury and hypermobility	356	16–50 (Mean: 25.9)			7
Kaiser Permanente ACLR registry	Regional (USA)	2005–present	Bojcic et al. [18] (CC)	ACL injury	1486	(Mean: 28, SD: 11)	KOOS✗	Before surgery, after surgery: 12, 24, 60 months	8
			Inacio et al. [126] (OC)	ACL injury	636	<14–50+ (Mean: 26, IQR: 18.7–36)			7
Cleveland ACL Registry	Hospital (USA)	(PD: 1991–1999)	Spindler et al. [127] (CC)	ACL injury	651	(Mean: 24, SD: 8)	KOOS✗ WOMAC✗ IKDC✗	Once	8
Multiligament Knee Injury Registry	Hospital (USA)	(PD: 2004–2014)	Woodmass et al. [128] (OC)	Multi-ligament injury	23	15–59 (Mean: 26)	IKDC✗ WOMAC✗ Lysholm✗	After surgery: 3, 12, 24 months	8
			Woodmass et al. [129] (OC)		20	16–52			8
International Global Surgical Registry	International		Duerr et al. [130] (OC)	ACL injury	287	12–60 (Mean: 27 SD: 11.8)	VAS✓ RAND-HSI✗ MARS✗ KOOS✗	Before surgery, after surgery: 12, 24 months.	7
Hospital ACL Registry	Hospital (Ireland)	(PD: 2014–2016)	Hurley et al. [131] (OC)	ACLR	126	(Mean: 22.3, SD: 5.2)	MARS✗ IKDC✗ CKRS✗ ACL-RSI✗	Before surgery, after surgery: 6, 9, 12, 24 months	7
Hospital ACL Registry	Hospital (USA)	(PD: 2000–2007	Barrett et al. [132] (OC)	ACL rupture	417 (37%)	12–59 (Mean: 17/39)	VAS✓ Lysholm✗ Tegner✗	Before surgery, after surgery: 3, 6, 9, 12, 18, 24 months	7
Hip Preservation Registries									
Hip Arthroscopy Registry	Hospital (USA)	(PD: 2008–2012)	Hartigan et al. [133] (OC)	Arthroscopy for femoro-acetabular impingement	78	14–39 (Mean: 23)	mHHS✗ NAHS✗ HOS-ADL✓ HOS-SSS✓ VAS✓	Before surgery, after surgery: 24 months	8
ANCHOR PAO Database	Regional (USA)	(PD: 2008–2012)	Stambough et al. [134] (CC)	PAO	117	9–35	UCLA✗ HOOS✗ SF12✗	Before surgery, after surgery	8
Hip Resurfacing Database	Hospital (UK)	(PD: 1999–2001)	Maclean et al. [135] (OC)	Hip resurfacing	143	12–30 (Mean: 21)	OHS✗	Before surgery, after surgery at 1.5 month intervals until discharge	8
Hospital Hip Database	Hospital (USA)	(PD: 2013–2017)	Pun et al. [136] (OC)	Reverse PAO for FAI	34	12–41	WOMAC✗ mHHS✗	Before surgery, after surgery	7

Key: # Study refers to more than one registry, ✓ = PROM validated for age range in study, ✗ = age range of study is outside validated range of PROM, ACL: anterior cruciate ligament, CC: case control study, CKRS: Cincinnati Knee Rating System, HOOS: Hip Disability and Osteoarthritis Outcome Score, HOS-ADL: Hip Outcome Score—Activities of Daily Living, HOS-SSS: Hip Outcome Score—Sport-Specific Subscale, IKDC: International Knee Documentation Committee, KOOS: Knee Injury and Osteoarthritis Outcome Score, K-SES: Knee Self-Efficacy Scale, MARS: Marx Activity Rating Scale, mHHS: Modified Harris Hip Score, NAHS: Non-Arthritic Hip Score, OC: observational cohort study, OHS: Oxford Hip Score, PAS: Physical Activity Scale, PD: published data, RAND-HSI: RAND Health Status Inventory, RTS: Return to Sport Questionnaire, SF12: Short Form-12, SF36: Short Form 36, UCLA: University of California Los Angeles Activity Scale, UK: United Kingdom, USA: United States of America, VAS: Visual Analogue Scale, WOMAC: Western Ontario and McMaster Universities Osteoarthritis Index.

**Table 4:** Registries reporting a small minority of paediatric patients (<33%).

Registry	Scope: Hospital/Regional/National/International (Nation)	Years Active	Publications (Type of Study)	Diagnostic Inclusion	Number of Patients in Publication (% of Registry)	Patient Age Range (Years) in Publication (Mean and SD or Median)	PROMs Used	Frequency of PROM Collection	Risk of Bias (0–9)
Lower Limb Arthroplasty Registries									
Oswestry International Arthroplasty Registry	International	1997–2002	Aulakh et al. [137] (OC)	Hip resurfacing	4535	13+	mHHS✗	Before surgery and after surgery: annually	7
			Aulakh et al. [138] (OC)		4535	13–88 (Mean: 52.6)			7
			Aulakh et al. [139] (CC)	RA and OA	178 (4)	16–67 (Mean: 43)			9
			Aulakh et al. [140] (CC)	Hip resurfacing	192	(Mean: 42/43)			8
Hospital Arthroplasty Registry	Hospital (Scotland)	2005–2009	Cowie et al. [141] (OC)	Hip arthroplasty	239	17–64 (Mean: 55.2, SD: 7.2)	UMWPAR✗	Before and after surgery	8
Multi-Centre Hip Arthroplasty Registry	National (France)	2010- (PD: 2010–2011)	Delaunay et al. [142] (OC)	Primary THA	2107	17–104	OHS✗	Once: At time of revision surgery	7
NZ Joint Registry	National (New Zealand)	1999–current (PD: 1998–2017)	Devane et al. [143] (OC)	THA	17,831 (25)	15–100 (Mean: 67)	OHS✗ OKS✗ MOxFQ✗	After surgery: 6, 60 months	7
			Hooper et al. [144] (OC)	THA, TKA	1165	15–100			8
			#Jeyaseelan et al. [145] (OC)	TAA	1502	32–96 (Mean: 66)			
			Pearse et al. [146] (OC)	TKA	16,403	8–100			8
			Rothwell et al. [147] (OC)	THA, TKA	7420 (24)	15–100			8
Orthovault (Hospital THR Registry)	Hospital (USA)	2001–2013	Gaillard et al. [148] (CC)	THA	3046	11–78	UCLA✗ VAS✓	After surgery: 1.5 months, annual	8
Elective Orthopaedic Centre (TKA)	Regional (UK)	2005–2008	Judge et al. [149] (OC)	TKA	1991	17–96	EQ5D✓ OKS✗	Before surgery, After surgery: 6 months	8
2 Hospital THA Registry	Regional (USA)	2006–2011	Delanois et al. [150] (OC)	Hip arthroplasty	35	14–88	mHHS✗	After surgery: 6 weeks, 3, 6, 12 months, every year.	7
Australian Joint Registry	National (Australia)	-2017	#Jeyaseelan et al. [145] (OC)	Ankle arthroplasty	2448	20–94	-	-	-
National Joint Registry (UK)	National (UK)	-2017	#Jeyaseelan et al. [145] (OC)	Ankle arthroplasty	4687	17–93	-	-	-
Swedish Ankle Registry	National (Sweden)	2016–2017	#Jeyaseelan et al. [145] (OC)	Ankle arthroplasty	66	16+	SEFAS✗ EQ5d✓		-
Hospital THA Database	Hospital (Scotland)	1990–1995	Kiran et al. [151] (OC)	Hip arthroplasty	100	16–55	VAS✓	After surgery: 36, 60, 120 months	7
Hospital THA Registry	Hospital (USA)	1996–2006	Le duff et al. [152] (CC)	Hip arthroplasty	125 and 533	14–78	SF12✓ UCLA✗	Before surgery, after surgery: 4, 12 months, annual	8
Swedish hip Arthroplasty Registry	National (Sweden)	2002–present (PD: 2002–2012)	Nemes et al. [153] (OC)	Hip arthroplasty	56,062	15–97	EQ5D✗ VAS✓	Before surgery, After surgery: 12 72, 120 months	7
			Rolfson et al. [154] (OC)		34,960	16–84			8
Hip Arthroplasty Hospital Registry	Hospital (USA)	(PD: 2000–2015)	Makarewich et al. [155] (CC)	Hip arthroplasty	1504	12–30 (younger group) 60–92 (older group)	PROMIS✓	Before surgery, after surgery: 12, 24, 60 months	8
Ireland THA	Hospital (Ireland)	2005–present (PD: 2005–2007)	Sheridan et al. [156] (OC)	Hip arthroplasty	1553	15–92	WOMAC✗	After surgery: 6, 24, 60, 120 months	7
Hospital Arthroplasty Registry	Hospital (Norway)	(PD: 2010–2012)	Winther et al. [157] (OC)	Hip or knee arthroplasty	1069	17–90	EQ5D✓ HOOS✗ KOOS✓ vNRS✓	Before surgery, after surgery: 2–3, 12 months	7
Hospital Hip Arthroplasty Registry	Hospital (Spain)	(PD: 2003–2008)	Ribas et al. [158] (OC)	Hip arthroplasty	450	16–69 (Mean: 47)	WOMAC✗	Before surgery, after surgery: 1, 3, 6 months, annually.	7
Joint Replacement Registry	Hospital (USA)	(PD: 2006–2008)	Wang et al. [159] (OC)	Hip arthroplasty	255	15–87 (Mean: 59, SD: 15)	WOMAC✗	Before surgery, after surgery: 3, 12 months	7
PG Database	National (USA)	(PD: 2009–2015)	Chughtai et al. [160] (OC)	Hip arthroplasty	692	15–91 (Mean: 62)	WOMAC✗ SF36✗ SF12✓ UCLA✗ VAS✓ PG survey✗	Once	7
			Delanois et al. [161] (CC)	Hip arthroplasty	692	15–91			7
			Patel et al. [162] (OC)	Hip arthroplasty	692	15–91			8
			Gwam et al. [163] (CC)	Joint arthroplasty	1454	15–92			9
National NHS PROMS	National (UK)	(PD: 2009–2011)	Lim et al. [164] (OC)	Hip arthroplasty	92,253	14–100 (Mean: 67, SD: 11)	OHS✗ (not specified)	Before surgery, After surgery: 6 months	7
Knee Ligament Registries:									
Swedish National Knee Ligament Register	National (Sweden)	2005–present (PD: 2004–2017)	Ageberg et al. [165] (CC)	ACL reconstruction and/or PCL reconstruction	5255	8–67	KOOS✗ EQ5D✗	Before surgery, after surgery: 12, 24, 60, 120 months	9
			Barenius et al. [166] (OC) Bergerson et al. [167] (OC)		3556 21,910	<18–>55 (not further specified) 15–71			7 7
			Desai at al [168] (OC)		22,699	7–74 (Median: 24)			7
			#Granan et al. [169] (OC)		7331	(Median: 25)			8
			Hamrin Senorski et al. [170] (OC)		6889	13–49			7
			Hamrin Senorski et al. [171] (OC)		13,636	13–49			8
			Hamrin Senorski et al. [172] (OC)		874	6–58			8
			Kraus Schmitz et al. [173] (OC)		26,014	7–74 (Mean: 26.8/31.4)			7
			Kvist et al. [174] (CC)		23,744 (100%)	(Mean: 26 (F), 28 (M))			8
			#Owesen et al. [175](OC)		1287	8–66			8
			Reinholdsson et al. [176] (CC)		3588	9–65			8
			Sandon et al. [177] (OC)		1661	(Mean: 23.5)			7
			Snaebjornsson et al. [178] (CC)		2240	13–67			9
			Svantesson et al. [179] (CC)		1014	13–49			8
			Svantesson et al. [180] (OC)		622	(Mean: 29.7)			7
			Svantesson et al. [181] (CC) Thorolfsson et al. [182] (OC)		22,460 2848 (7%)	13–50+ 5–35			7 8
			#Ulstein et al. [183] (OC)		8470	9–69			7
Norwegian National Knee Ligament Registry	National (Norway)	2004–present (PD: 2004–2013)	Årøen et al. [184] (CC)	ACL or PCL	9720	12–67	KOOS✗	Before surgery, after surgery: 24, 60, 120 months	9
			Engen et al. [185] (CC)	Focal cartilage defects	58	10–55 (Mean: 29.8)			8
			Granan et al. [186] (OC)	ACLR	3475	17–40			8
			Granan et al. [187] (OC)	ACL and PCL injuries	2793	12–67			8
			#Granan et al. [169] (OC)	ACLR	7331	(Median: 25)			8
			#Grindem et al. [118] (CC)		84	16–40 (Mean: 25.3, SD: 7.2)			8
			Hjermundrud et al. [188] (CC)	Full thickness cartilage lesion	90	15–39			9
			Ingelsrud [189] (OC)	ACLR	1197	(Mean: 28/29)			8
			#Magnussen et al. [113] (OC)		4928/5720 (not stated)	(Median: 27 IQ 19–36)			9
			#Owesen et al. [175] (OC)	PCLR	1287	14–67			8
			#Ulstein [183] (OC)	ACLR	8470	9–69			7
Danish Knee Ligament Registry	National (Denmark)	2005–present	#Granan et al. [169] (OC)	Knee ligament injury	7331	10–71	KOOS✗	After surgery: 12 months	8
			Nissen et al. [190] (CC)	Revision ACLR	1619	15–59			9
		(PD: 2004–2013)	Owesen et al. [175] (OC)	PCLR	1287	15–60			8
Project ACL	Regional (Sweden)	2014–present	Beischer et al. [191] (CC) Högberg et al. [192] (OC) Piussi et al. [193] (CC)	ACL injury ACLR ACLR	655 137 641	(Mean: 22, SD: 4) (Mean: 25, SD: 8) (Mean: 24.8, SD: 7.6)	ACL-RSI✗ K-SES✗ Tegner✗ KOOS✓	After surgery: 2.5, 4, 8, 12 months	8 8 7
Surgeon Knee Registry	Hospital (USA)	Not reported	Lubowitz et al. [194] (CC)	ACL injury	128	13–66 (Mean: 38)	QWB✓		8
ACLR Hospital Database	Hospital (USA)	2007–2014	Miller et al. [195] (OC)	ACL injury	660	12–68	KOS-ADL✓ vNRS✓		8
Hospital ACL Registry	Hospital (Serbia)	2012–2013	Ninkovic et al. [196] (OC)	ACL injury	185	16–55	KOOS✓ Lysholm✗		7
Hospital ACLR Registry	Hospital (Singapore)	2013–2016	Panjwani et al. [197] (OC)	ACLR	270	15–52 (Mean: 25)	KOOS✗ SF 36✗		8
ACL Treatment Registry	Regional (USA)	2011–2015	Centeno et al. [198] (OC)	ACL injury	29	15–65 (Mean: 35)	LEFS✗ IKDC✗ VAS✓ SANE✗		7
Hospital ACLR Registry	Hospital (Singapore)	(PD: 2009–2012)	Singh et al. [199] (CC)	ACL injury	264	(Mean: 24, SD: 6)	Lysholm✗ Tegner✗		7
Hospital ACL Registry	Hospital (USA)	(PD: 2015–present)	Bedeir et al. [200] (CC)	ACL injury	221	(IQ range: 17–37)	IKDC✗ KOOS✗ MARS✗ RTS✗	Before surgery, after surgery: 6, 12, 24 months	7
Hospital ACL Registry	Hospital (Austria)	(PD: 2010–2016)	Runer et al. [201] (OC)	ACLR	875	(Mean: 31, 29, 31)	Lysholm✗ Tegner✗ VAS✓	Before surgery, after surgery: 6, 12, 24 months	7
Hospital ACL Registry	Hospital (USA)	(PD: 2015–2018)	Duncan et al. [202] (CC)	ACLR	184	15–50	ACL-RSI✗	Before surgery, at return to sport	7
Hospital ACL Registry	Hospital (USA)	(PD: 2016–2020)	Hazzard et al. [203] (CC)	ACLR	264	15–45 (Mean: 30, SD: 7)	VAS✓ KOOS✗ IKDC✗ Tegner✗ Lysholm✗ SANE✗ RAND-HSI✗	Before surgery, 6, 12, 24 months	7
Hip Preservation									
Danish National Patient Registry/Hospital Database	Hospital (Denmark)	2010–present (PD: 2004–2017)	Larsen et al. [204] (OC)	PAO	1126	13–59 (Median: 32)	HOOS✗ VAS✓	Before surgery, after surgery: 6, 24, 60, 120 months	8
Hip Arthroscopy Registry	Hospital (USA)	(PD: 2012–2015)	Leong et al. [205] (OC)	Hip arthroscopy	700	12–73 (Mean: 33.2)	HOS-ADL✗	Before surgery, after surgery: 24 months	8
Hip Arthroscopy registry	Hospital (NZ)	(PD: 2012–2016)	Brick et al. [206] (CC)	Hip arthroscopy for femoro-acetabular impingement	634	13–59 (Mean: 35, SD: 12)	iHOT-12✗ NAHS✗ HOOS✗ VAS✓	Before surgery, after surgery: 24 months	8
Hip Preservation Registry	Hospital (USA)	(PD: 2006–2013)	Okoroafor et al. [207] (OC)	PAO for acetabular dysplasia	70	14–47 (Mean: 25)	UCLA✗ mHHS✗ WOMAC✗	Before surgery, after surgery at follow-up	8
Non-Arthroplasty Hip Registry	National (UK)	2002–present (PD: 2013–2015)	Humphrey et al. [208] (OC)	Non-arthroplasty hip surgery	381	15–70	iHOT✗ Eq5d✗	Before surgery, after surgery: 6 months	7
Hip Arthroscopy Database	Hospital (USA)	(PD: 2009–2014)	Tjong et al. [209] (OC)	Femoro-acetabular impingement and labral tears	86	17–59 (Mean: 38)	iHOT-12✗ mHHS✗	Once: after surgery: 24 months	8
Non-Arthroplasty Hip Registry	National (UK)	2012–present (PD: 2013–2015)	Maempel et al. [210] (OC)	Femoro-acetabular impingement	88	15–57	EQ5D✗ iHOT12✗ VAS✓	Before surgery, after surgery: 12 months	8
Hospital Registry	Hospital (Canada)	2005–present (PD: 2005–2020)	Ibrahim et al. [211] (OC) Ibrahim et al. [212] (OC) Laboudie et al. [213] (OC)	Femoro-acetabular impingement PAO PAO	88 67 15	17–49 16–54 16–40	HOOS✗ WOMAC✗ UCLA✗ SF12✓	Before surgery, after surgery at final follow-up	8
NY Hip Preservation Registry	Hospital (USA)	2010-present (PD: 2010–2015)	Ricciardi et al. [214] (CC)	Femoro-acetabular impingement	1765 (100%)	10–75	mHHS✗ iHOT-33✗ HOS✗	Before surgery, after surgery: 6, 12, 24, 36 months	8
			Ricciardi et al. [215] (CC)	PAO	93	12–43			8
			Ricciardi et al. [216] (CC)	PAO	77	12–43			7
			Ricciardi et al. [217] (OC)	Previous pelvic surgery	147	11–76			7
Arthroscopy Database	Hospital (USA)	(PD: 2009–2011)	Redmond et al. [218] (CC)	Hip arthroscopy	893	13–76 (Mean: 38, SD: 14)	mHHS✗ NAHS✗ HOS✓ VAS✓	Once: before surgery	8
Single Surgeon FAIS Registry	Hospital (USA)	(PD: 2010–2015)	Chenard et al. [219] (CC)	Femoro-acetabular impingement syndrome	318 (68%)	14–70	mHHS✗ NAHS✗	Before surgery, after surgery: 1, 3, 6, 12, 24 months	9
Hip Surgery Registry	Hospital (USA)	(PD: 2007–2010)	Heyworth et al. [220] (OC)	PAO	41	13–41 (Mean: 26)	HOOS✗ UCLA✗	Before surgery, after surgery at follow-up (until 12 months)	8
Ligamentum Teres Reconstruction Registry	Hospital (USA)	(PD: 2012–2016)	Rosinsky et al. [221] (OC)	Ligamentum teres reconstruction	676	17–43 (Mean: 30)	NAHS✗ mHHS✗ VAS✓	Before surgery, after surgery annually	8
Hospital PAO registry	Hospital (USA)	(PD: 2008–2015)	Wyles et al. [222] (CC)	PAO	221 (75%)	13–48	UCLA✗ HOOS✗ WOMAC✗ SF12✗	Before surgery, after surgery: 12, 24, 60 months	7
Hospital Hip Registry	Hospital (Ireland)	(PD: 2008–2010)	Carton et al. [223] (OC)	Femoro-acetabular impingement	138	15–54	mHHS✗ UCLA✗ SF36✗ WOMAC✗	Before surgery, After surgery: 120 months	7
Spine Registries									
NorSpine	National (Norway)	2013–2016	Polak et al. [224] (CC)	Spine surgery	1750	16–87 (Mean: 50)	Eq5d✓ vNRS✓ ODI✗	Before surgery, after surgery: 3, 12 months	8
Spine Tango Registry	National (Germany)	2012-	Neukamp et al. [225] (OC)	Spine surgery	2510	17–93 (Mean: 51.2, SD: 15.4)	VAS✓	After surgery: 3, 6 months	7
SweSpine	National (Sweden)	1993/2006–present (PD 2013–2017)	Beck et al. [226] (OC)	Spine surgery	92	15–59	vNRS✓ ODI✗ EQ5D3L✗ SRS22r✓ VAS✓ SF36✗	Before surgery, after surgery annually	8
		(PD 2006–2013)	Charalampidis et al. [227] (OC)	Idiopathic scoliosis	328	10–20			7
		(PD: 2006–2009)	Ersberg et al. [228] (OC)	Scoliosis	211	9–20			8
		(PD 1998–2017)	Lagerback et al. [229] (CC)	Lumbar disc herniation	4537	(Means of two groups: 17 and 33)			9
British Spine Registry	National (United Kingdom)	2012–present	Gardner er al [230] (OC)	AIS and spine deformity	16,439 (100%)	10–18 years of those reported in publication, age not reported in 50%	SRS22✓	Before surgery, after surgery: 1.5, 6, 12, 24, 60, 84, 120 months	7
Thoracolumbar Injury Registry	Regional (Austria)	(PD 1994–1996)	Knop et al. [231] (OC)	Thoracolumbar injuries	1168	9–95 (Mean: 47)	VAS✓	Before surgery, after surgery	7
Other Knee Registries									
Knee Registry	Hospital (USA)	(PD: 2006–2008)	Wang et al. [232] (CC)	Osteochondral allograft transplant	75 (4%)	14–62 (Mean: 34.9)	SF 36✗ IKDC✗ KOS-ADL✓ CKRS✗ MARS✗	Before surgery, after surgery	8
Cartilage Transplant Registry	Hospital (USA)	(PD: 1983–2011)	Gracitelli et al. [233] (OC)	Osteochondral allograft transplant	27	14–64 (Mean: 33)	IKDC✗ KS-F✗ KOOS✗	Before surgery, after surgery	8
			Briggs et al. [234] (OC)		55 (6%)	15–67 (Mean: 42)			7
			Cameron et al. [235] (OC)		28	12–47			6
Cartilage Repair Registry	Regional (USA)	Not stated	Mandelbaum et al. [236] (OC)	Autologous chondrocyte implantation	40 (not specified)	16–48 (Mean: 37)	CKRS✗	Before surgery, after surgery annually	8
AMIC Registry	Regional (USA)	2005–present	Gille et al. [237] (OC)	Autologous matrix-induced chondrogenesis	57 (not specified)	17–61 (Mean: 37.3)	Lysholm✗ VAS✓	Before surgery, after surgery: 12, 24 months	7
Hospital Registry	Hospital (USA)	(PD: 2007–2015)	Ogura et al. [238] (OC)	Autologous chondrocyte implantation	242	14–58 (Mean: 31.4/34)	KOOS✗ IKDC✗ Lysholm✗ SF12✓	Before surgery, after surgery	7
Patella Instability registry	Hospital (USA)	(PD: 2012–2016)	Khazi et al. [239] (CC)	Patellofemoral stabilization	60	(Means: 22 and 30, SD: 10)	KOOS✗ Kujala✓	After injury: immediate, 6, 24 months	7
Patellofemoral Database	Hospital (UK)	(PD: 2013–2018)	Sharma et al. [240] (OC)	Patellar instability	202	12–51 (Mean: 24.2)	IKDC✗ EQ5D✗ Kujala✗	Before surgery, after surgery 12 months	7
Trauma Registries									
Japanese Database of Orthopaedic Trauma	National (Japan)	(PD: 2015–2019)	Kurozumi et al. [241] (CC)	Severe lower limb open fractures	45	7–95	LEFS✗ SF-8✗	Before surgery, after surgery	8
Victorian Orthopaedic Trauma Outcomes Registry	Regional (Australia)	2003–present (PD: 2009–2016)	Andrew et al. [242] (OC)	Sport-related injuries	366	15–74	SF12✓ vNRS✓ EQ5D3L✗ ”RTW” questions✗ HADS✗ PTSD Checklist✗ IEQ✗ ASES✗ SSV✗ VAS✓	After injury: (variable) discharge, 6, 12, 24 months	7
			Devlin et al. [243] (OC)	On-road collision injuries	6186	16–75+ (Mean: 37.8–48.8)			8
			Diacon et al. [244] (CC)	Multi-trauma with foot fractures	122	(Mean: 38)			8
			Ekegren et al. [245] (OC)	Hip fractures	291	17–64			8
			Ekegren et al. [246] (OC)	Hip fractures	507	17–64			8
			Ferguson et al. [247] (OC)	Tibial shaft fractures	60	16–77			7
			Fox et al. [248] (OC)	Surgical repair of Achilles tendon	204	17–83			8
			Giummarra et al. [249] (CC) Giummarra et al. [250] (CC)	Traumatic injury Unintential injury	732 Not individually reported	17–64 16–85+			7 8
			Hoogervorst et al. [251] (OC)	Fractured lower limb	111	16–60+			7
			Papakonstantinou et al. [252] (OC)	Proximal humerus fractures	306	16–80+			8
			Salipas et al. [253] (OC)	Medial clavicle fracture	68	16–94			7
			Urquhart et al. [254] (OC)	Orthopaedic trauma	1181	15–100			7
			Williamson et al. [255] (OC)	Orthopaedic trauma	1290	14–95			7
Hospital Trauma Registry	Hospital (Australia)	(PD: 2008–2015)	Hoskins et al. [256] (OC)	High-energy neck of femur fractures	32	15–50 (Mean: 38)	iHOT12✗ Eq5D✗	At follow-up (not specified)	7
Detroit Trauma Registry	Hospital (USA)	(PD: 2000–2011)	Vaidya et al. [257] (CC)	Low-velocity knee dislocations	19	15–74 (Mean: 30)	Tegner✗	At follow-up (not specified)	9
Swedish Fracture Registry	National (Sweden)	2011–present	Wennergren et al. [258]	Fractures	N/A	16–100+, 16–20 = 5%	EQ5D3L✓ SMFA✗	After surgery: immediate, 12 months	-
Pain Registries									
National Pain Registry	National (UK)	(PD: 2010–2011)	Duncan et al. [19] (OC)	Acute pain	9748	0–100 (Mean: 57)	WBPQ✗	Once	6
PAINOUT	International	(PD: 2010–2013)	Zaslansky et al. [259] (CC)	Acute pain	14,334	16–unknown	IPO-Q✗	Before surgery, after surgery	7
			Chapman et al [260] (CC)		9272				6
Tumour Registries									
Tumour Database	Hospital (UK)	Not reported	Maclean et al. [261] (OC)	Humerus tumour	8	16–78, 1 of 8 patients <18	TESS✓	At follow-up	8
Tumour Registry	Hospital (Canada)	(PD: Prior to 2001)	Beadel et al. [262] (CC)	Pelvic tumour	26	16–64 (Mean: 41)	TESS✓	At follow-up (not specified)	7
Tumour Registry	Hospital (India)	(PD: 2011–2017)	Gulia et al. [263] (OC)	Giant cell tumour	12	15–41 (Mean: 29)	PRWE✗	Once	8
Shoulder Registries									
Norwegian Shoulder Instability Registry	National (Norway)	(PD: 2008–2009)	Blomquist et al. [264] (OC)	Shoulder stabilisation	464	10–74	WOSI✗	Before surgery, after surgery: 12, 24, 36 months	7
Shoulder Arthroplasty Registry	Hospital (USA)	(PD: 1991–2017)	Hackett et al. [265] (OC)	Shoulder arthroplasty	983	17–87	SST✗	Before surgery	7
MOON Shoulder Instability registry	Regional (USA)	(PD: 2012–2016)	Duchman et al. [266] (OC)	Shoulder stabilisation surgery	545	12–99 (Mean: 24.1, SD: 8.7)	SF36✗ WOSI✗ ASES✗ SAS✗	Before surgery	8
Hospital Shoulder Registry	Hospital (USA)	(PD: 2017–2019)	Vadhera et al. [267] (CC)	Bankart and rotator cuff repair	488	(Mean: 29.3, SD: 12.5)	PROMIS✓ ASES✗ SANE✗ SF12✓ RAND-HIS✗	Before surgery, after surgery	7
Foot/Ankle Registries									
National Ankle Reconstruction Database	National (Canada)	(PD: 2002–2014)	Gagné et al. [268] (OC)	Ankle reconstruction	194	17–54 (Mean: 47, SD: 7.2)	SF36✓	Before surgery, after surgery: 6, 12 months, annual	7
Hallux Valgus registry	Hospital (Singapore)	(PD: 2007–2015)	Law et al. [269] (CC)	Hallux valgus surgery	721	14–83 (Mean: 59, SD: 8)	VAS✓ SF36✓	After surgery: 6, 24 months	9
Other Registries									
Global Surgical Registry	International	Not reported	Ryu et al. [20] (OC)	Arthroscopy knee procedure	1725	Not specified (includes 18% patients below 18 years)	IKDC✗	Not reported	6
Dutch Hospital Registry	National (The Netherlands)	(PD: 2003–2010)	Borghans et al. [270] (OC)	Hospital-wide including orthopaedic surgery	10,2815	0–65+	COPS✗	Once	7
Sports Medicine Registry	Hospital (USA)	(PD: 2017)	Lizzio et al. [271] (OC)	Sports medicine clinic attendance	581	11–95	PROMIS✓	Once	8
Maryland Orthopaedic Registry	Hospital (USA)	(PD: 2015–2018)	Sajak et al. [272] (OC)	Post-op ortho surgery	1269	17+	PROMIS✓ IKDC✗ ASES✗ bMHQ✗ MODEMS-E✗ IPAQ✗ Tegner✗ MARS✗	Before surgery, after surgery: 0.5 months	7
Allograft Registry	Hospital (USA)	(PD: 2013–2020)	Cook et al. [273] (OC) Cook et al. [274] (OC) Oladeji et al. [275] (OC)	Osteochondral allograft knee Osteochondral allograft knee Osteochondral allograft hip	25 76 10	13–51 15–69 17–49	PROMIS✓ IKDC✗ SANE✗ VAS✓ HOOS✗	Before surgery, after surgery: 0.5, 1.5, 3, 6, 12 months, annually	8 8 7

Key: # study refers to more than one registry, ✓ = PROM validated for age range in study, ✗ = age range of study is outside validated range of PROM, ACL anterior cruciate ligament, ACLR: anterior cruciate ligament reconstruction, ACL-RSI: Anterior Cruciate Ligament—Return to Sport after Injury Scale, ASES: American Shoulder and Elbow Surgeons Shoulder Score, bMHQ: Brief Manchester Hand Questionnaire, CC: case control study, CKRS: Cincinnati Knee Rating System, COPS: Core Questionnaire for the Assessment of Patient Satisfaction, EQ5D: EuroQol 5 Dimensions, EQ5D3L: EuroQol 5 Dimensions 3 Levels, HADS: Hospital Anxiety and Depression Scale, HOOS: Hip Disability and Osteoarthritis Outcome Score, HOS: Hip Outcome Score, HOS-ADL: Hip Outcome Score—Activities of Daily Living, IEQ: Injustice Experience Questionnaire, iHOT: International Hip Outcome Tool, IKDC: International Knee Documentation Committee, IPAQ: International Physical Activity Questionnaire, IPO-Q: International Pain Outcome—Questionnaire, KOOS: Knee Injury and Osteoarthritis Outcome Score, KOS-ADL: Knee Outcome Survey Activities of Daily Living Scale, K-SES: Knee Self-Efficacy Scale, KS-F: Knee Society–Function, LEFS: Lower Extremity Functional Scale, MARS: Marx Activity Rating Scale, mHHS: Modified Harris Hip Score, MODEMS-E: Musculoskeletal Outcomes Data Evaluation and Management System—Expectations, MoxFQ: Manchester Oxford Foot and Ankle Questionnaire, NAHS: Non-Arthritic Hip Score, NHS: National Health Service, NZ: New Zealand, OA: osteoarthritis, OC: observational cohort study, ODI: Oswestry Disability Index, OHS: Oxford Hip Score, OKS: Oxford Knee Score, PAO: Periacetabular Osteotomy, PCL: posterior cruciate ligament, PCLR: posterior cruciate ligament reconstruction, PD: published data, PG: Press Ganey, PROMIS^®^: Patient Reported Outcomes Measurement Information System^®^, PRWE: Patient-Rated Wrist Evaluation, PTSD: post-traumatic stress disorder, QWB: quality of well-being, RA: rheumatoid arthritis, RAND_HSI: RAND Health Status Inventory, RTW: return to work, SANE: Single Assessment Numeric Evaluation, SAS: Shoulder Activity Score, SEFAS: Self-Reported Foot Ankle Score, SF12: Short Form 12, SF36: Short Form 36, SMFA: Short Musculoskeletal Function Assessment, SRS: Scoliosis Research Society, SST: Simple Shoulder Test, SSV: Subjective Shoulder Value, TAA: total ankle arthroplasty, TESS: Toronto Extremity Salvage Score, THA: total hip arthroplasty, TKA: total knee arthroplasty, UCLA: University of California Los Angeles Activity Scale, UMWPAR: Unspecified Measure of Work, Physical Activity, and Restriction, USA: United States of America, UK: United Kingdom, VAS: Visual Analogue Scale, vNRS: Numerical Rating Scale, WBPQ: Web-Based Pain Questionnaire, WOMAC: Western Ontario and McMaster Universities Arthritis Index, WOSI: Western Ontario Shoulder Instability Index.

**Table 5:** PROMs used among paediatric patients in orthopaedic registries.

PROM	Frequency of Use	Acceptable Psychometric Properties	Validated in Adults	Paediatric Validation Ages
Single Question (3)				
VAS [276]	23	Yes	Yes	5+ years
vNRS [277]	8	Yes	Yes	8+ years
SANE [278]	4	Yes	Yes	
Generic (21)				
PODCI [279]	2	Yes	No	2–18 years
PROMIS [280]	6	Yes	Yes	5–18 years
PedsQL [281]	1	Yes	No	2–18 years
WeeFIM [282]	1	Yes	No	6 months–7 years
CHQ [283]	1	Yes	No	5–18 years
QWB [284]	1	Yes	Yes	7+ years
SF36 [285]	9	Yes	Yes	16+ years
SF12 [286]	12	Yes	Yes	14+ years
EQ5D/EQ5D3L [287]	10/4	Yes	Yes	16+ years
PAS [288]	1	Yes	Yes	
ODI [289]	3	Yes	Yes	
RAND-HIS [290]	3	Yes	Yes	
LEFS [291]	2	Yes	Yes	
IPO-Q [292]	1	Yes	Yes	
COPS [293]	1	Yes	Yes	
MODEMS-E [294]	1	Yes	Yes	
IPAQ [295]	1	Yes	Yes	
UMWPAR [141]	1	No	No	
PG Survey [296]	1	Yes	Yes	
RTW [249]	1	No	No	
SMFA [297]	1	Yes	Yes	
Spine (4)				
SRS30 [298]/24 [299]/22 [300]/22r [301]/29/7	3/2/10/6/1/1	Yes	Yes	10+ years
EOSQ24 [302]	2	Yes	No	0–18 years
BIDQ [303]	1	Yes	Yes	14+ years
SAQ [304]	1	Yes	Yes	6+ years
Hip (8)				
HOS [305](ADL)(SSS)	5	No	Yes	13+ years
mHHS [306]	14	Yes	Yes	
iHOT 12 [307]/33 [308]	4/3	Yes	Yes	
NAHS [309]	5	Yes	Yes	
UCLA [310,311]	9	Yes	Yes	
HOOS [312]	9	Yes	Yes	
OHS [313]	4	Yes	Yes	
WOMAC [314]	12	Yes	Yes	
Knee (14)				
Pedi IKDC [315]	1	Yes	No	10–18 years
Lysholm [316]	10	Yes	Yes	
Tegner [317]	9	Yes	Yes	
KOOS [318]	19	Yes	Yes	16+ years
MARS [319]	7	Yes	Yes	
IKDC [320]	17	Yes	Yes	
RTS [117]	4	No	No	
K-SES [288]	2	Yes	Yes	16+ years
ACL-RSI [321]	3	Yes	Yes	16+ years
KOS-ADL [322]	2	Yes	Yes	12+ years
KS-F [323]	1	Yes	Yes	
OKS [324]	2	Yes	Yes	
Kujala [325]	2	Yes	Yes	15+ years
CKRS [326]	3	Yes	Yes	17+ years
Foot (2)				
MOxFQ [327]	1	Yes	Yes	
SEFAS [328]	1	Yes	Yes	
Upper Limb (7)				
SSV [329]	1	Yes	Yes	
PRWE [330]	1	Yes	Yes	
WOSI [331]	2	Yes	Yes	
SST [332]	1	Yes	Yes	
BMHQ [333]	1	Yes	Yes	
SAS [334]	1	Yes	Yes	
ASES [335]	4	Yes	Yes	
Other (13)				
TESS [336]	2	Yes	Yes	16+ years
CRIES [337]	1	Yes	No	8–18 years
CPCHILD [338]	1	Yes	No	5–18 years
RSBC [339]	1	Yes	No	0+
RS: SSI [340]	1	Yes	No	0+
GFAQ [341]	1	Yes	No	3+ years
Custom DMD [101]	1	No	No	
Custom SMA [21]	1	No	No	
Custom Morquio [102]	1	No	No	
HADS [342]	1	Yes	Yes	
PTSD Checklist [343]	1	Yes	Yes	
IEQ [344]	1	Yes	Yes	
WBPQ [19]	1	No	No	

**Table 6:** Types of registries that include patients under the age of 18 years.

Type of Registry	Number (%)	100% Paediatric	>50% Paediatric	<50% Paediatric	<33% Paediatric
Knee Ligament	27 (21)	0	1	12	14
Lower Limb Joint Arthroplasty	21 (16)	0	1	0	20
Spine	21 (16)	1	15	0	5
Hip Preservation	21 (16)	0	2	4	15
Other Knee	8 (6)	0	1	0	7
Trauma	6 (5)	1	0	0	5
Rare Disease	4 (3)	0	4	0	0
Shoulder	4 (3)	0	0	0	4
Pain	4 (3)	0	2	0	2
Tumour	3 (2)	0	0	0	3
Foot/Ankle	2 (2)	0	0	0	2
Other	7 (5)	1	1	0	5
TOTAL	128	3	27	16	82

## Data Availability

A systematic review protocol was made and registered at the International Prospective Register of Systematic Reviews (PROSPERO). The protocol can be accessed at: https://www.crd.york.ac.uk/prospero/display_record.php?ID=CRD42021215364 (accessed on 13 August 2023).

## Supplementary Materials

The following supporting information can be downloaded at: https://www.mdpi.com/article/10.3390/children10091552/s1, Text S1: Complete Search Strategy; Text S2: Newcastle-Ottawa Quality Assessment Form for Cohort Studies & Case-Control.
