# Supplementary material for: Evaluating the Use of PROMs in Paediatric Orthopaedic Registries
Source: Children (Basel). 2023 Sep 14;10(9):1552. doi: 10.3390/children10091552 (PMC10528097; doi:10.3390/children10091552)
Supplement: Supplementary file 1 [file children-10-01552-s001.zip › children-2584703-Supplementary.pdf]

children MDPI

Supplementary Text S1: Complete Search Strategy

The following search strategy was used for each database:

Medline Search Terms

1. Pediatrics/
2. paediatric*.mp
3. pediatric*.mp
4. Child/
5. child*.mp
6. Infant/
7. Infant*.mp
8. Young Adult
9. young adult*.mp
10. Adolescent/
11. adolescen*.mp
12. teen*.mp
13. young people*.mp
14. young person*.mp
15. 1 or 2 or 3 or 4 or 5 or 6 or 7 or 8 or 9 or 10 or 11 or 12 or 13 or 14
16. Orthopedics/
17. orthopaedic*.mp
18. orthopedic*.mp
19. implantable medical device*.mp
20. Legg-Calve-Perthes Disease/
21. perthes*.mp
22. legg-calve-perthes*.mp
23. Hip Dislocation/
24. Hip Dislocation, Congenital/
25. hip 1islocate*.mp
26. hip dysplasia*.mp
27. developmental dysplasia of the hip*.mp
28. Limb Deformities, Congenital/ or Hand Deformities, Congenital/ or Upper Extremity Deformities, Congenital/
29. (congenital limb deformit* or congenital hand deformit* or congenital upper extremity deformit*).mp
30. Slipped Capital Femoral Epiphyses/
31. slipped capital femoral epiphyses.mp
32. scfe.mp
33. Clubfoot/
34. clubfoot*.mp
35. Talipes/
36. congenital talipes equinovarus.mp
37. Anterior Cruciate Ligament Injuries/
38. Anterior Cruciate Ligament Reconstruction.
39. (anterior cruciate ligament and (injur* or reconstruction*)).mp
40. Knee Injuries/
41. Knee injur*.mp
42. Scoliosis/
43. scoliosis.mp
44. neuromuscular scoliosis.mp
45. idiopathic scoliosis.mp
46. 16 or 17 or 18 or 19 or 20 or 21 or 22 or 23 or 24 or 25 or 26 or 27 or28 or 29 or 30 or 31 or 32 or 33 or 34 or 35 or 36 or 37 or 38 or 39 or 40 or 41 or 42 or 43 or 44 or 45
47. Registries/
48. registr*.mp
49. repository*.mp
50. Database Management Systems/
51. register.mp
52. orthopaedic regist*.mp
53. orthopedic regist*.mp
54. arthroplasty regist*.mp
55. registries in orthop*.m_titl.
56. 46 or 47 or 48 or 49 or 50 or 51 or 52 or 53 or 54 or 55
57. Patient Reported Outcome Measures/
58. patient reported outcome measure*.mp
59. proms.tw
60. "Surveys and Questionnaires"/
61. survey*.mp
62. questionnaire*.mp
63. proxy survey*.mp
64. patient reported measure*.mp
65. 57 or 58 or 59 or 60 or 61 or 62 or 63 or 64
66. 15 and 46 and 56 and 65

Embase Search Terms

1. P?ediatric*.mp
2. Pediatrics/
3. Child/
4. Child*.mp
5. Infant/
6. Infant*.mp
7. Young adult/
8. Young adult*.mp
9. Adolescent/
10. Adolescen*.mp
11. Teen*.mp
12. Young people*.mp
13. Young person*.mp
14. 1 or 2 or 3 or 4 or 5 or 6 or 7 or 8 or 9 or 10 or 11 or 12 or 13
15. Orthopedics/
16. Orthop?edic*.mp
17. Implantable medical device.mp
18. Pethes disease/
19. Perthes*.mp
20. Legg-calve-perthes*.mp
21. Hip dislocation/
22. Hip 2islocate*.mp
23. Hip dysplasia/
24. Hip dysplas*.mp
25. Congenital hip dislocation/
26. Congenital hip 2islocate*.mp
27. Developmental dysplasia of the hip.mp
28. Limb malformation/ or limb deformity/ or congenital malformation/
29. Limb deformit*.mp
30. Limb malform*.mp
31. Congenital malform*.mp
32. Slipped capital femoral epiphysis/
33. Slipped capital femoral epiphys*.mp
34. Scfe*.mp
35. Clubfoot/
36. Clubfoot*.mp
37. Talipes.mp
38. Congenital talipes equinovarus.mp
39. Anterior cruciate ligament/
40. Anterior cruciate ligament*.mp
41. Anterior cruciate ligament reconstruction/
42. Anterior cruciate ligament reconstruct*.mp
43. Anterior cruciate ligament injury/
44. Anterior cruciate ligament injur*.mp
45. Knee injury/
46. Knee injur*.mp
47. Scoliosis/
48. Scoliosis*.mp
49. Idiopathic scoliosis/
50. Idiopathic scoliosis*.mp
51. Adolescent idiopathic scoliosis/
52. Adolescent idiopathic scoliosis*.mp
53. Neuromuscular scoliosis.mp
54. 15 or 16 or 17 or 18 or 19 or 20 or 21 or 22 or 23 or 24 or 25 or 26 or 27 or 28 or 29 or 30 or 31 or 32 or 33 or 34 or 35 or 36 or 37 or 38 or 39 or 40 or 41 or 42 or 43 or 44 or 45 or 46 or 47 or 48 or 49 or 50 or 51 or 52 or 53
55. Register/
56. Register*.mp
57. Registr*.mp
58. Database management system/
59. Database management system*.mp
60. Orthop?edic regist*.mp
61. P?ediatric regist*.mp
62. 55 or 56 or 57 or 58 or 59 or 60 or 61
63. Patient-reported outcome/
64. Patient-reported outcome*.mp
65. Proms*.mp
66. Health survey/
67. Health survey*.mp
68. Questionnaire/
69. Questionnaire*.mp
70. Proxy survey.mp
71. Proxy questionnaire.mp
72. Patient reported measure.mp
73. 63 or 64 or 65 or 66 or 67 or 68 or 69 or 70 or 71 or 72
74. 14 and 54 and 62 and 73

Web of Science Search Terms

1. P?ediatric*

2. Child
3. Infant
4. Young adult
5. Adolescen*
6. Teen*
7. “young people”
8. “young person”
9. 1 or 2 or 3 or 4 or 5 or 6 or 7 or 8
10. orthop?edic
11. “implantable medical device*”
12. Perthes
13. “hip dislocation”
14. “hip dysplasia”
15. “limb deformities”
16. “congenital limb deformities”
17. “Slipped capital femoral epiphysis”
18. “SCFE”
19. “congenital talipes equinovarus”
20. “CTEV”
21. Clubfoot
22. Talipes
23. “Anterior Cruciate ligament”
24. “knee injury”
25. “scoliosis”
26. “Neuromuscular scoliosis”
27. “idiopathic scoliosis”
28. 10 or 11 or 12 or 13 or 14 or 15 or 16 or 17 or 18 or 19 or 20 or 21 or 22 or 23 or 24 or 25 or 26 or 27
29. Registr*
30. Repositor*
31. Register*
32. Database
33. 29 or 30 or 31 or 32
34. “patient reported outcome measure”
35. “prom”
36. Survey
37. Questionnaire
38. “Patient reported measure”
39. “proxy survey”
40. “proxy questionnaire”
41. 29 or 30 or 31 or 32 or 33 or 34 or 35 or 36 or 37 or 38 or 39 or 40
42. 9 and 28 and 33 and 41

Scopus Search Terms

1. P?ediatric*
2. Child
3. Infant
4. “young adult”
5. Adolescen*
6. Teen*
7. “young people”
8. “young person”
9. 1 or 2 or 3 or 4 or 5 or 6 or 7 or 8
10. Orthop?edic
11. “Implantable medical device*”
12. Perthes
13. “Hip dislocation”
14. “Hip dysplasia”
15. “limb deformities”
16. “congenital limb deformities”
17. “Slipped capital femoral epiphysis”
18. “SCFE”
19. “clubfoot”
20. “Congenital talipes equinovarus”
21. talipes
22. “CTEV”
23. “Anterior Cruciate ligament”
24. “knee injury”
25. “Scoliosis”
26. “neuromuscular Scoliosis”
27. “idiopathic Scoliosis”
28. 10 or 11 or 12 or 13 or 14 or 15 or 16 or 17 or 18 or 19 or 20 or 21 or 22 or 23 or 24 or 25 or 26 or 27
29. Registr*
30. Repositor*
31. Register*
32. Database*
33. 29 or 30 or 31 or 32
34. “patient reported outcome measure”
35. “prom”

36. Survey
37. Questionnaire
38. "Patient reported measure"
39. "proxy survey"
40. "proxy questionnaire"
41. 34 or 35 or 36 or 37 or 38 or 39 or 40
42. 9 and 28 and 33 and 41

Cinahl Search Terms

1. "paediatric"
2. "pediatric"
3. (MH "Child+")
4. "child"
5. (MH "Infant+")
6. "infant"
7. (MH "Young Adult")
8. "young adult"
9. (MH "Adolescence+")
10. "adolescent"
11. "teenager"
12. "young people"
13. "young person"
14. 1 or 2 or 3 or 4 or 5 or 6 or 7 or 8 or 9 or 10 or 11 or 12 or 13
15. (MH "Orthopedics")
16. "orthopedics"
17. (MH "Orthopedic Surgey+")
18. "orthopaedics"
19. "implantable medical device"
20. (MH "Legg-Perthes Disease")
21. "perthes"
22. (MH "Hip Dislocation")
23. (MH Hip Dislocation Congenital")
24. "hip dysplasia"
25. (MH "Limb Deformities, Congenital+")
26. (MH "Upper Extremity Deformities, Congenital+")
27. (MH "Lower Extremity Deformities, Congenital+")
28. (MH "Epiphyses, Slipped")
29. "slipped capital femoral epiphysis"
30. (MH "Clubfoot")
31. "clubfoot"
32. "talipes"
33. 15 or 16 or 17 or 18 or 19 or 20 or 21 or 22 or 23 or 24 or 25 or 26 or 27 or 28 or 29 or 30 or 31 or 32
34. (MH "Registries, Disease+")
35. (MH "Registries, Implant")
36. (MH "Registries, Trauma")
37. (MH "Registries, Personnel")
38. "registry"
39. "register"
40. "database"
41. (MH "Clinical Data Repository")
42. "repository"
43. "orthopaedic registry"
44. (MH "Orthopedic Fixation Devices+")
45. 34 or 35 or 36 or 37 or 38 or 39 or 40 or 41 or 42 or 43 or 44
46. (MH "Patient-Reported Outcomes+")
47. "patient reported outcome measure"
48. (MH "Surveys+")
49. (MH "Survey Research")
50. (MH "Survey Questionnaires+")
51. "questionnaires"
52. (MH "Structured Questionnaires")
53. "proms"
54. "patient reported measure"
55. 46 or 47 or 48 or 49 or 50 or 51 or 52 or 53 or 54

Google Scholar Search Terms

1. p?ediatric*
2. Child
3. Infant
4. "young adult"
5. adolescen*
6. teen*
7. "young people"
8. "young person"
9. 1 or 2 or 3 or 4 or 5 or 6 or 7 or 8
10. Orthop?edic
11. "implantable medical device*"
12. Perthes

13. “hip dislocation”
14. “hip dysplasia”
15. “limb deformities”
16. “congenital limb deformities”
17. “slipped capital femoral epiphysis”
18. “SCFE”
19. Clubfoot
20. Talipes
21. “congenital talipes”
22. 10 or 11 or 12 or 13 or 14 or 15 or 16 or 17 or 18 or 19 or 20 or 21
23. Registr*
24. Repositor*
25. Register*
26. Database*
27. 23 or 24 or 25 or 26
28. “patient reported outcome measure”
29. “prom”
30. Survey
31. Questionnaire
32. “Patient reported measure”
33. “Proxy survey”
34. “proxy questionnaire”
35. 28 or 29 or 30 or 31 or 32 or 33 or 34

9 and 22 and 27 and 35

Supplementary Text S2

Newcastle-Ottawa Quality Assessment Form for Cohort Studies & Case-Control

* indicates 1 point towards total score

Shading indicates Case-Control Studies

Study	Selection				Comparability (** possible)	Outcome (Cohort) Exposure (Case-Control)			Total Score
Cohort Studies	Representa-tiveness of the exposed cohort	Selection of the non-exposed co-hort	Ascertainment of exposure	Demonstration that outcome of in-terest was not pre-sent at start of study	Comparability of cohorts on the ba-sis of the design or analysis controlled for confounders	Assessment of out-come Case control	Was follow-up long enough for outcomes to occur	Adequacy of fol-low-up	
Case-Control Studies	Is the case def-inition ade-quate?	Representative-ness of the cases	Selection of con-trols	Definition of con-trols	Comparability of cases and controls on the basis of the design or analysis	Ascertainment of exposure	Same method of ascertainment for cases and controls	Non-response rate	
Ageberg et al 2010 [179]	*	*	*	*	**	*	*		8
Andrew et al 2014 [331]	*	N/A	*	*	**	*	*		7
Årøen et al 2012 [180]	*	*	*	*	**	*	*	*	9
Aulakh et al 2014 [236]	*	N/A	*	*	*	*	*	*	7
Aulakh et al 2015 [237]	*	N/A	*	*	*	*	*	*	7
Aulakh et al 2011 [238]	*	*	*	*	**	*	*	*	9
Aulakh et al 2010 [239]	*	*	*	*	*	*	*	*	8
Badin et al 2023[54]	*	*	*	*	*	*	*		7
Bae et al 2018 [24]	*	N/A	*	*	**	*	*	*	8
Barenius et al 2013 [259]	*	N/A	*	*	**	*	*		7
Barrett et al 2011 [231]	*	*	*	*	*	*	*		7
Bastrom et al 2022 [32]	*	*	*	*	**	*	*		8
Bastrom et al 2019 [33]	*	*	*	*	*	*	*	*	8

Bastrom et al 2015 [17]	*	N/A	*	*	*	*	*		6
Bastrom et al 2013 [34]	*	N/A	*	*	**	*	*		7
Bauer et al 2019 [60]	*	*	*	*	*	*	*		7
Beadel et al 2005 [349]	*	*	*	*	*	*	*		7
Beck et al 2020 [316]	*	N/A	*	*	**	*	*	*	8
Bedeir et al 2019 [290]	*	*	*	*	*	*	*		7
Beischer et al 2019 [281]	*	*	*	*	**	*	*		8
Belter et al 2021 [21]	*	N/A	*	*	*	*	N/A		5
Benes et al 2023 [35]	*	N/A	*	*	*	*	*	*	7
Bennett et al 2013 [36]	*	N/A	*	*	*	*	*	*	7
Bennett et al 2017 [37]	*	*	*	*	*	*	*		7
Bergerson et al 2022[260]	*	*	*	*	*	*	*		7
Blomquist et al 2012 [351]	*	N/A	*	*	**	*	*		7
Bojcic et al 2014 [18]	*	*	*	*	*	*	*		6
Borghans et al 2012 [357]	*	N/A	*	*	**	*	*		7
Bourassa-Moreau et al 2010 [93]	*	*	*	*	*	*	*		7
Boykin et al 2013 [29]	*	*	*	*	**	*	*		8
Brick et al 2020 [296]	*	*	*	*	**	*	*		8
Briggs et al 2015 [323]	*	N/A	*	*	**	*	*		7
Buckland et al 2019 [38]	*		*	*	**	*	*	*	8
Cameron et al 2015 [324]	*	N/A	*	*	*	*	*		6
Campbell et al 2020 [61]	*	*	*	*	**	*		*	8
Carreon et al 2010[79]	*	N/A	*	*	**	*	*		7
Carton et al 2021[313]	*	N/A	*	*	*	*	*	*	7
Centeno et al 2018 [288]	*	N/A	*	*	**	*	*		7
Chapman et al 2013 [347]	*	*	*	*	**	*			7
Charalampidis et al 2018 [188]	*	N/A	*	*	**	*	*		7

Chenard et al 2020 [309]	*	*	*	*	**	*	*	*	9
Chughtai et al 2018 [254]	*	N/A	*	*	**	*	*		7
Cook et al 2022 [360]	*	N/A	*	*	**	*	*	*	8
Cook et al 2023 [361]	*	N/A	*	*	**	*	*	*	8
Cowie et al 2013 [128]	*	N/A	*	*	**	*	*		7
Crawford et al 2013 [80]	*	*	*	*	*	*	*		7
Daley et al 2023 [25]	*	N/A	*	*	**	*		*	7
Delanois et al 2018 [255]	*	*	*	*	*	*	*		7
Delanois et al 2017 [245]	*	N/A	*	*	**	*	*		7
Delaunay et al 2013 [183]	*	N/A	*	*	*	*	*	*	7
Desai et al 2014 [261]	*	N/A	*	*	**	*	*		7
Devane et al 2013 [186]	*	N/A	*	*	**	*	*		7
Devlin et al 2019 [332]	*	N/A	*	*	**	*	*	*	8
Diacon et al 2019 [333]	*	*	*	*	**	*	*		8
Diebo et al 2019 [94]	*	N/A	*	*	*	*	*	*	7
Djurasovic et al 2018 [89]	*	*		*	**	*	*		7
Downs et al 2009 [100]	*	N/A	*	*	**	*	*		7
Duchman et al 2018 [353]	*	N/A	*	*	**	*	*	*	8
Duerr et al 2019 [229]	*	N/A	*	*	**	*	*		7
Duncan et al 2014 [19]	*	N/A	*	*	*	*	*		6
Duncan et al 2023[292]	*	*	*	*	*	*	*		7
Dunn et al 2010 [212]	*	N/A	*	*	**	*	*	*	8
Eguia et al 2020 [55]	*	N/A	*	*	**	*	*		7
Ekegren et al 2017 [194]	*	N/A	*	*	**	*	*	*	8
Ekegren et al 2016 [195]	*	N/A	*	*	**	*	*	*	8
Engen et al 2017 [276]	*	*	*	*	**	*	*		8
Ersberg et al 2013 [317]	*	N/A	*	*	**	*	*	*	8

Failla et al 2016 [204]	*	*	*	*	**	*	*		8
Fausett et al 2023 [182]	*	N/A	*	*	**	*	*		7
Ferguson et al 2008 [334]	*	N/A	*	*	**	*	*		7
Fletcher et al	*	*	*	*	*	*	*	*	8
Fox et al 2016 [335]	*	N/A	*	*	**	*	*	*	8
Gagné et al 2018 [355]	*	N/A	*	*	**	*	*		7
Gaillard et al 2017 [243]	*	*	*	*	*	*	*	*	8
Ganesh et al 2007 [103]	*	N/A	*	*	*	*	*	*	7
Gardner et al 2021[319]	*	N/A	*	*	**	*	*		7
Gilat et al 2021 [107]	*	*	*	*	*	*	*		7
Gille et al 2013 [326]	*	N/A	*	*	**	*	*		7
Giummarra et al 2017 [336]	*	*	*	*	*	*	*		7
Giummarra et al 2022 [337]	*	N/A	*	*	**	*	*	*	8
Godzik et al 2015 [95]	*	*	*	*	*	*	*		7
Gomez et al 2022 [62]	*	N/A	*	*	**	*	*		7
Gracitelli et al 2015 [322]	*	N/A	*	*	**	*	*		7
Granan et al 2009 [277]	*	N/A	*	*	**	*	*	*	8
Granan et al 2008 [278]	*	N/A	*	*	**	*	*	*	8
Granan et al 2009 [262]	*	N/A	*	*	**	*	*	*	8
Grindem et al 2015 [217]	*	*	*	*	*	*	*	*	8
Gulia et al 2019 [350]	*	N/A	*	*	**	*	*	*	8
Gwam et al 2017 [257]	*	*	*	*	**	*	*	*	9
Hackett et al 2018 [352]	*	N/A	*	*	**	*	*		7
Hamrin Senorski et al 2017 [223]	*	N/A	*	*	**	*	*	*	8
Hamrin Senorski et al 2017 [263]	*	N/A	*	*	**	*	*		7
Hamrin Senorski et al 2017 [264]	*	N/A	*	*	**	*	*	*	8
Hamrin Senorski et al 2018 [205]	*	N/A	*	*	**	*	*	*	8

Hartigan et al 2016 [232]	*	N/A	*	*	**	*	*	*	8
Hazzard et al 2023 [293]	*	*	*	*	*	*	*		7
Heffernan et al 2022 [63]	*	*	*	*	*	*	*		8
Helenius et al 2019 [64]	*	*	*	*	**	*	*	*	9
Helenius et al 2019 [65]	*	*	*	*	**	*	*	*	9
Henstenburg et al 2023 [66]	*	N/A	*	*	**	*	*	*	8
Heyworth et al 2016 [310]	*	N/A	*	*	**	*	*	*	8
Hjermundrud et al 2010 [279]	*	*	*	*	**	*	*	*	9
Högberg et al 2023 [282]	*	N/A	*	*	**	*	*	*	8
Hoogervorst et al 2021 [338]	*	N/A	*	*	**	*	*		7
Hooper et al 2009 [240]	*	N/A	*	*	**	*	*	*	8
Hoskins et al 2019 [343]	*	N/A	*	*	**	*	*		7
Humphrey et al 2018 [301]	*	N/A	*	*	**	*	*		7
Hughes et al 2021 [39]	*	*	*	*	*	*	*		7
Hurley et al 2021 [230]	*	N/A	*	*	*	*	*	*	7
Ibrahim et al 2018 [301]	*	N/A	*	*	**	*	*	*	8
Ibrahim et al 2021[302]	*	N/A	*	*	**	*	*		7
Inacio et al 2014 [225]	*	N/A	*	*	*	*	*	*	7
Ingelsrud et al 2015 [280]	*	N/A	*	*	**	*	*	*	8
Jain et al 2015 [40]	*	N/A	*	*	*	*	*	*	7
Jain et al 2017 [56]	*	N/A	*	*	*	*	*	*	7
Jeyaseelan et al 2019 [185]	(multiple registries, unable to score)								
Judge et al 2012 [244]	*	N/A	*	*	**	*	*	*	8
Kelly et al 2019 [41]	*	N/A	*	*	**	*	*		7
Khazi et al 2020 [328]	*	*	*	*	**	*	*		8
Kiran et al 2018 [246]	*	N/A	*	*	**	*	*		7
Knop et al 2006 [320]	*	N/A	*	*	**	*	*		7

Kraus Schmitz et al 2019 [265]	*	N/A	*	*	**	*	*		7
Kurozumi et al 2022 [330]	*	*	*	*	*	*	*	*	8
Kvist et al 2014 [266]	*	*	*	*	**	*	*		8
Laboudie et al 2022 [303]	*	N/A	*	*	*	*	*	*	7
Lagerback et al 2019 [318]	*	*	*	*	**	*	*	*	9
Landman et al 2011[82]	*	N/A	*	*	**	*	*		7
Lark et al 2013 [42]	*	*	*	*	*	*	*	*	8
Larsen et al 2020 [294]	*	N/A	*	*	**	*	*	*	8
Law et al 2020 [356]	*	*	*	*	**	*	*	*	9
Le Duff et al 2007 [247]	*	*	*	*	**	*	*		8
Leong et al 2018 [295]	*	N/A	*	*	**	*	*	*	8
Li et al 2020 [98]	*	N/A	*	*	**	*	*		7
Lim et al 2015 [258]	*	N/A	*	*	**	*	*		7
Lindanger et al 2019 [154]	*	*	*	*	**	*	*	*	9
Lizzio et al 2019 [358]	*	N/A	*	*	**	*	*	*	8
Lonner et al 2013 [43]	*	N/A	*	*	**	*	*		7
Louer et al 2019 [44]	*	N/A	*	*	*	*	*	*	7
Lubowitz et al 2011 [284]	*	*	*	*	*	*	*	*	8
Luhmann et al 2012 [83]	*	*	*	*	*	*	*	*	8
Maclean et al 2015 [234]	*		*	*	**	*	*	*	8
Maclean et al 2017 [348]	*	N/A	*	*	**	*	*	*	8
Maempel et al 2018 [300]	*	N/A	*	*	**	*	*	*	8
Magnussen et al 2010 [213]	*	N/A	*	*	**	*	*	*	9
Makarewich et al 2018 [249]	*	*	*	*	**	*	*		8
Mandelbaum et al 2007 [325]	*	N/A	*	*	**	*	*	*	8
Mather et al 2013 [214]	*	N/A	*	*	**	*	*	*	8
Matsumoto et al 2022 [67]	*	*	*	*	*	*	*	*	8

Matsumoto et al 2020 [68]	*	*	*	*	*	*	*		7
Matsumoto et al 2021 [69]	*	N/A	*	*	**	*	*	*	8
Matsumoto et al 2021 [70]	*	*	*	*	**	*	*	*	9
Mcmulkin et al 2016 [105]	*	*	*	*	*	*	*	*	8
Mens et al 2022 [96]	*	N/A	*	*	*	*	*	*	7
Messner et al 2020 [23]	*	N/A	*	*	**	*	*	*	8
Miller et al 2017 [285]	*	N/A	*	*	**	*	*	*	8
Miller et al 2020 [57]	*	N/A	*	*	**	*	*	*	8
Miyanji et al 2018 [58]	*	N/A	*	*	*	*	*	*	7
Miyanji et al 2015 [92]	*		*	*	*	*	*	*	8
Montano et al 2007 [102]	*	N/A	*	*	**	*	*	*	8
Negrini et al 2011 [91]	*	N/A	*	*	**	*	*	*	8
Nemani et al 2015 [90]	*	N/A	*	*	*	*	*	*	7
Nemes et al 2015 [184]	*	N/A	*	*	**	*	*		7
Neukamp et al 2013 [315]	*	N/A	*	*	**	*	*		7
Newton et al 2020 [45]	*	N/A	*	*	**	*	*		7
Newton et al 2022 [46]	*	*	*	*	*	*	*	*	8
Ninkovic et al 2015 [286]	*	N/A	*	*	**	*	*		7
Nissen et al 2018 [181]	*	*	*	*	**	*	*	*	9
Nossov et al 2022 [71]	*	*	*	*	*	*	*	*	8
Nwachukwu et al 2017 [30]	*	N/A	*	*	**	*	*	*	8
Nwachukwu et al 2017 [218]	*	N/A	*	*	**	*	*	*	8
Nwachukwu et al 2017 [219]	*	N/A	*	*	**	*	*		7
Nwachukwu et al 2017 [220]	*	N/A	*	*	**	*	*		7
Ogura et al 2020 [327]	*	N/A	*	*	*	*	*	*	7
Ohashi et al 2020 [47]	*	*	*	*	**	*	*		8
Okoroafor et al 2019 [297]	*	N/A	*	*	**	*	*	*	8

Oladeji et al 2018 [362]	*	N/A	*	*	**	*	*		7
Owesen et al 2017 [267]	*	N/A	*	*	**	*	*	*	8
Pallante et al 2020 [28]	*	N/A	*	*	**	*	*	*	8
Panjwani et al 2019 [287]	*	N/A	*	*	**	*	*	*	8
Papakonstantinou et al 2017 [339]	*	N/A	*	*	**	*	*	*	8
Patel et al 2017 [256]	*	N/A	*	*	**	*	*	*	8
Pearse et al 2010 [241]	*	N/A	*	*	**	*	*	*	8
Phillips et al 2019 [48]	*	*	*	*	**	*	*		8
Piussi et al 2022 [283]	*	*	*	*	*	*	*		7
Polak et al 2020 [314]	*	*	*	*	**	*	*		8
Pun et al 2021[235]									
Qiu et al 2020 [22]	*	N/A	*	*	**	*	*		7
Rahardja et al 2021 [210]	*	N/A	*	*	**	*	*	*	8
Ramirez et al 2022[72]	*	N/A	*	*	*	*	*	*	7
Ramkumar et al 2019 [215]	*	N/A	*	*	**	*	*	*	8
Ramo et al 2021[73]	*	N/A	*	*	*	*	*	*	7
Randsborg et al 2022 [221]	*	N/A	*	*	**	*	*		7
Rauck et al 2021 [222]	*	*	*	*	*	*	*		7
Redmond et al 2017 [308]	*		*	*	*	*	*	*	8
Reinholdsson et al 2017 [268]	*	*	*	*	**	*	*		8
Ribas et al 2014 [252]	*	N/A	*	*	**	*	*		7
Ricciardi et al 2015 [304]	*	*	*	*	**	*	*		8
Ricciardi et al 2017 [305]	*	*	*	*	**	*	*		8
Ricciardi et al 2017 [306]	*	*		*	**	*	*		7
Ricciardi et al 2014 [307]	*	N/A	*	*	**	*	*		7
Roberts et al 2011[85]	*	N/A	*	*	**	*	*		7
Rolfson et al 2011 [248]	*	N/A	*	*	**	*	*	*	8

Rosinsky et al 2020 [311]	*	N/A	*	*	**	*	*	*	8
Rothwell et al 2010 [242]	*	N/A	*	*	**	*	*	*	8
Roye et al 2020 [74]	*	N/A	*	*	**	*	*	*	8
Roye et al 2022 [75]	*	*	*	*	*	*	*		7
Runer et al 2020 [291]	*	N/A	*	*	**	*	*		7
Ryu et al 2023 [20]	*	N/A	*	*	**	*			6
Sajak et al 2020 [359]	*	N/A	*	*	**	*	*		7
Salipas et al 2016 [340]	*	N/A	*	*	**	*	*		7
Saarinen et al 2022 [76]	*	*	*	*	*	*	*		7
Sanders et al 2010 [86]	*	*	*	*	*	*	*		7
Sandon et al 2020 [269]	*	N/A	*	*	**	*	*		7
Schorling et al 2020 [101]	*	N/A	*	*	**	*	*	*	8
Schulz et al 2014 [49]	*	N/A	*	*	*	*	*	*	7
Schwartz et al 2004 [106]	*	N/A	*	*	**	*	*	*	8
Segal et al 2020 [50]	*	*	*	*	*	*	*		7
Serbin et al 2023[31]	*	N/A	*	*	**	*	*		7
Sharma et al 2021 [329]	*	N/A	*	*	*	*	*	*	7
Shaw et al 2022 [77]	*	N/A	*	*	**	*	*	*	8
Sheridan et al 2019 [250]	*	N/A	*	*	*	*	*	*	7
Sieberg et al 2013 [84]	*	N/A	*	*	**	*	*		8
Singh et al 2018 [289]	*	*	*	*	*	*	*		7
Singla et al 2014 [51]	*	*	*	*	*	*	*		7
Snaebjornsson e al 2017 [270]	*	*	*	*	**	*	*	*	9
Spindler et al 2003 [226]	*	*	*	*	*	*	*	*	8
Stambough et al 2015 [233]	*	*	*	*	*	*	*	*	8
Stone et al 2023 [52]	*	N/A	*	*	**	*	*		7
Sundemo et al 2022 [224]	*	*	*	*	*	*	*		7

Svantesson et al 2020 [271]	*	*	*	*	**	*	*		8
Svantesson et al 2020 [272]	*	N/A	*	*	**	*	*		7
Svantessonet al 2017[273]	*	*	*	*	*	*	*		7
Theologis et al 2015[87]	*	*	*	*	*	*	*		7
Thomas et al 2023[99]	*	N/A	*	*	*	*	*	*	7
Thorolfsson et al 2022[274]	*	*	*	*	*	*	*	*	8
Tiplady et al 2023[211]	*	*	*	*	*	*	*	*	8
Tjong et al 2017[299]	*	N/A	*	*	**	*	*	*	8
Ulstein et al 2018[275]	*	N/A	*	*	**	*	*		7
Upasani et al 2008[53]	*	N/A	*	*	*	*	*	*	7
Urquhart et al 2006[341]	*	N/A	*	*	**	*	*		7
Vadhera et al 2021[354]	*	*	*	*	*	*	*		7
Vaidya et al 2015[344]	*	*	*	*	**	*	*	*	9
Verhofste et al 2021[78]	*	*	*	*	*	*	*		7
Vivas et al 2020[59]	*	N/A	*	*	**	*	*	*	8
Wall et al 2020[26]	*	N/A	*	*	**	*	*	*	8
Wall et al 2021[27]	*	*	*	*	*	*	*		7
Wang et al 2017[321]	*	*	*	*	**	*	*		8
Wang et al 2012[253]	*	N/A	*	*	**	*	*		7
Wennergren et al 2015[345]	(Registry report, no results)								
Williamson et al 2009[342]	*	N/A	*	*	**	*	*		7
Winther et al 2015[251]	*	N/A	*	*	**	*	*		7
Woodmass et al 2018[227]	*	N/A	*	*	**	*	*	*	8
Woodmass et al 2018[228]	*	N/A	*	*	**	*	*	*	8
Wright et al 2007[216]	*	N/A	*	*	**	*	*	*	8
Wyles et al 2019[312]	*	*	*	*	*	*	*		7
Zaslansky et al 2018[346]	*	*	*	*	*	*	*	N/A	7

Zebracki et al 2013[88]	*	N/A	*	*	*	*	*	*	7
Zhang et al 2023[104]	*	*	*	*	*	*	*		7
Zhu et al 2017[97]	*	*	*	*	*	*	*		7
